# Quality of life, functional impairment and continuous performance task event‐related potentials (ERPs) in young adults with ADHD and autism: A twin study

**DOI:** 10.1002/jcv2.12090

**Published:** 2022-07-10

**Authors:** Ümit Aydin, Simone J. Capp, Charlotte Tye, Emma Colvert, Alex Lau‐Zhu, Frühling Rijsdijk, Jason Palmer, Gráinne McLoughlin

**Affiliations:** ^1^ Social, Genetic & Developmental Psychiatry Centre Institute of Psychiatry, Psychology & Neuroscience King's College London London UK; ^2^ Department of Child and Adolescent Psychiatry Institute of Psychiatry, Psychology & Neuroscience King's College London London UK; ^3^ Department of Psychology Institute of Psychiatry, Psychology & Neuroscience King's College London London UK; ^4^ Oxford Institute for Clinical Psychology Training and Research Medical Sciences Division University of Oxford Oxford UK; ^5^ Psychology Department Faculty of Social Sciences Anton de Kom University Paramaribo Suriname; ^6^ Endowed Research Department of Clinical Neuroengineering Global Center for Medical Engineering and Informatics Osaka University Suita Japan

**Keywords:** ADHD, autism, event‐related potentials, functional impairment, quality of life, twin study, young adults

## Abstract

**Background:**

Young adulthood is a key developmental period for understanding outcomes of childhood onset attention‐deficit/hyperactivity disorder (ADHD) and autism. Measurement of functional impairment and quality of life (QoL) can provide important information on the real‐life challenges associated with these conditions. Event‐related potential (ERP) measures from the continuous performance task (CPT) have long been identified as altered in ADHD and autism but the role of these functions in the aetiological pathway to the disorders and associated impact on quality of life in young adulthood is unknown.

**Method:**

We investigated the relationships between ADHD and autism, functional impairment, quality of life, and ERP measures from the cued CPT (CPT‐OX) in a young adult twin sample (566 participants aged 22.43 ± 0.96 years old).

**Results:**

We observed significant phenotypic correlations between ADHD/autism and lower quality of life with specific genetic overlap between ADHD and physical health, psychological, and environmental aspects. We found significant phenotypic and genetic correlations between ADHD and functional impairment in all domains, as well as between autism and impairment in social functioning and lower impairment in risk‐taking. Both ADHD and autism were associated with attenuated amplitude of inhibitory and proactive control ERPs, with large genetic contributions to the overlap. We also found significant phenotypic correlations between these ERP measures and Weiss Functional Impairment Rating Scale (WFIRS) and QoL.

**Conclusion:**

This is the first study to investigate the phenotypic and genetic relationships between ADHD and autism, functional impairment, quality of life and ERP measures in young adulthood. Our findings could represent a step towards identifying ERP measures that are related to behaviour in the absence of overt symptoms.

## Graphical abstract

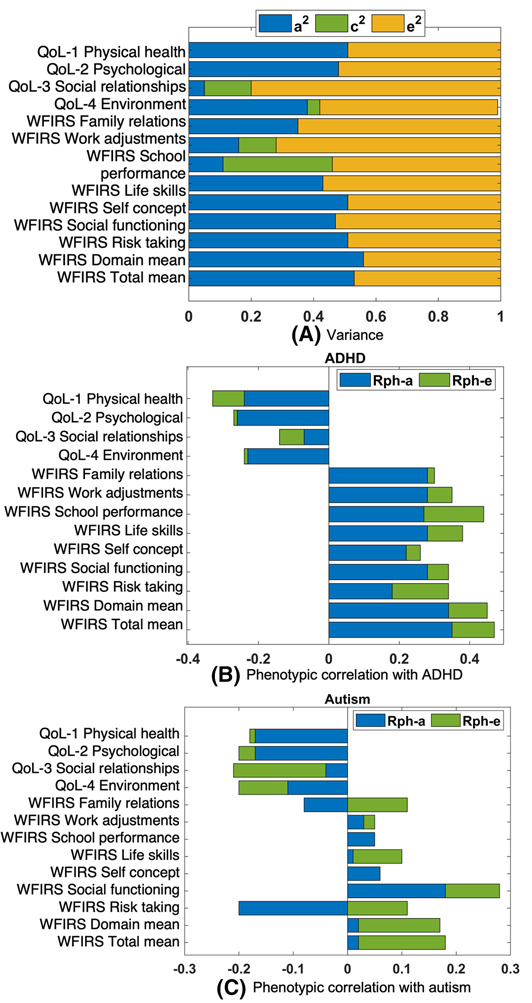

Young adulthood (ages 18–25) is a key developmental period for understanding adult outcomes of childhood onset ADHD and autism. We investigated the relationships between ADHD and autism, functional impairment, quality of life, and event‐related potentials from continuous performance task in a young adult twin sample. We observed significant associations accompanied with significant genetic and environmental overlaps in many cases.

**Key points**

- First study to investigate the phenotypic and genetic relationships between attention‐deficit/hyperactivity disorder (ADHD) and autism, functional impairment, quality of life (QoL), and event‐related potentials (ERPs) in a young adult twin sample.
- Decreased QoL in both ADHD and autism. ADHD is associated with increased functional impairment in all domains while autism is associated with higher/lower impairment in social‐functioning/risk‐taking.
- Both ADHD and autism are associated with abnormal ERPs time‐locked to inhibitory and cue stimuli with large genetic overlap in some. Significant associations between these ERP measures and functional impairment and QoL.

## INTRODUCTION

The window in development between adolescence and adulthood –young adulthood (ages 18–25) – is critical for understanding adult outcomes of childhood onset ADHD and autism (Lau‐Zhu et al., 2019). Although symptom‐based assessments are the ‘gold‐standard’ for clinical outcomes, symptoms may be distinct from the real‐life challenges faced by adults with ADHD and autism who are at higher risk of experiencing a range of behavioural and cognitive problems including poorer academic performance and lower employment levels (Davidson, 2008; Levy & Perry, 2011). In line with the emerging view of medicine as “health promoting” rather than “life preserving”, there has been a shift in focus in ADHD and autism research to quality of life (QoL), general wellbeing and day‐to‐day functioning beyond the clinical diagnosis (Danckaerts et al., 2010). Individuals who no longer have an ADHD diagnosis but retain some symptoms have been shown to have lower work productivity, QoL, and self‐esteem along with higher functional impairment in social life, family life/home responsibilities, and work/school (Pawaskar et al., 2020). Similarly, a meta‐analysis showed that autistic individuals experience significantly lower QoL than non‐autistic individuals as they transition into adulthood (van Heijst & Geurts, 2015).

The use of cognitive biomarkers to predict and track outcomes in ADHD and autism has the potential to improve clinical impact by providing quantitative measures for intervention planning and personalised treatment plans. A common strategy for the understanding of brain pathophysiology across neurodevelopmental conditions is to examine cognitive and neural dysfunction that is closely related to the core behavioral symptoms. Accordingly, a majority of such studies in ADHD in particular aim to address questions focused on selective or sustained attention, inhibitory control and effort allocation (Johnstone et al., 2013) and have identified consistent alterations in these and other executive functions (Willcutt et al., 2005). Similarly, broad executive function deficits have been reported in autism across development (Demetriou et al., 2018). Moreover, difficulties with executive function (self‐reported and neuropsychologically assessed) has been related to poorer QoL in both ADHD and autism (de Vries & Geurts, 2015; Stern et al., 2017).

One of the most studied tasks in neurodevelopment is the continuous performance task (CPT (Lau‐Zhu et al., 2019)). The cued version of the task (CPT‐OX (Doehnert et al., 2008; McLoughlin et al., 2010, 2011)) has the advantage of measuring the P3, or P300, in multiple contexts. When the P3 event‐related potential (ERP) is elicited by the no‐go stimulus, where a participant must refrain from making a prepotent or automated response, it is called the inhibition‐related or no‐go P3, and projects to frontal regions of the scalp (Fallgatter et al., 2002). The P3 in response to predictive cues, which is maximal at posterior scalp sites, represents proactive control (a preparatory process involved in optimally biasing attention to goal‐relevant information). The ‘cue’ and the ‘no‐go’ P3s have been consistently identified as attenuated in both autism and ADHD in both children and adults (Cui et al., 2017; Kaiser et al., 2020; Lau‐Zhu et al., 2019). Additional cue processing alterations in ADHD and autism are seen in the contingent negative variation (CNV), a frontocentral slow negative potential observed during the anticipatory interval after a cue stimulus (Kaiser et al., 2020; Tye et al., 2014).

A number of studies indicate a shared familial relationship between ERP measures from the CPT‐OX task and ADHD (Albrecht et al., 2008; McLoughlin et al., 2009; Michelini et al., 2021). However, these studies relied on classic family designs with non‐twin relationships (or without relatives with differing degrees of relatedness, e.g., cousins), and thus were unable to discriminate between genetic and environmental influences. Given the lack of such studies in the existing literature, the precise aetiology of the relationship between these disorders and ERP indices of the CPT‐OX is currently unknown. Twin studies provide a powerful way to delineate the aetiological architecture of cognitive changes associated with neurodevelopmental disorders (McLoughlin, Palmer, et al., 2014). Such an approach goes beyond simply estimating genetic and environmental contributions to single measures to examine shared genetic (or environmental) variance between cognitive/brain markers and behaviour.

In the present study, we investigate the aetiological relationships between ERP indices of the CPT‐OX and ADHD and autism diagnosis in 283 young adult twin pairs. We further examine the relationship between these measures and functional impairment and QoL. First, we investigated the relationship between QoL and the WFIRS and ADHD and autism in young adulthood. We expected decreased QoL and higher functional impairment for both ADHD and autism (De Groot, 2020; Mason et al., 2018; Quintero et al., 2019). Second, we investigated the relationship between CPT‐OX ERPs and ADHD and autism. Based on the findings from childhood, we expected broad differences in ERP measures from the CPT‐OX, particularly for ADHD. Finally, we investigated the association between the ERPs and functional impairment and QoL.

## MATERIALS AND METHODS

Throughout the manuscript we will be using both identity‐first language (i.e., “autistic person”) and person‐first language (i.e., “person with autism”) when referring to the individuals with high traits on these conditions.

### Study sample

Full ethical approval for the study was received from King's College London Psychiatry, Nursing and Midwifery Research Ethics Subcommittee (RESCMR‐16/17‐2673). Data were collected as part of the Individual Differences in EEG in young Adults Study (IDEAS). IDEAS participants were all recruited from the Twins Early Development Study (TEDS), a community sample of over 16,000 twin pairs born in England and Wales between 1994 and 1996 (Haworth et al., 2013; Rimfeld et al., 2019). The sample was enriched for high levels of autistic and/or ADHD traits based on childhood and adolescent measures (see Supporting Information). All participants contacted and recruited to the IDEAS study had an estimated IQ of 70 or above.

The selective recruitment strategy employed in this study, especially with regard to adolescent autistic and ADHD traits and estimated IQ, meant that while IDEAS was community‐based (rather than a clinical or convenience sample), it was not intended to be a whole community or community representative sample. The sample consisted of 556 participants (267 males) with an average age of 22.43 ± 0.96 years (119 Monozygotic (MZ) and 164 Dizygotic (DZ) pairs) (see full sample description in the Supporting Information and Table S1). Based on the Diagnostic Interview for ADHD in Adults 2.0 (DIVA‐2) and the Autism Diagnostic Observation Schedule‐2 (ADOS‐2), 111 participants met criteria for ADHD and 47 for autism (corresponding to a clinical diagnosis). Participants with a comorbid ADHD and autism diagnosis (16 participants) were included in both the ADHD and autism groups.

### Psychological measures

Prior to analyses, all data were cleaned and corrected for errors (see Supporting Information and (Capp et al., 2022)).

#### In‐person interviews/assessments

‐ ***Diagnostic Interview for ADHD in Adults 2.0 (DIVA‐2):*** a semi‐structured interview conducted by a trained investigator to assess ADHD symptoms (Kooij & Francken, 2010). We used the DSM‐5 diagnostic criteria for adult ADHD (recall of childhood onset of symptoms and five or more symptoms of inattention and/or hyperactivity/impulsivity that cause problems in more than one life domain) (American Psychiatric Association, 2013).
‐ ***Autism Diagnostic Observation Schedule‐2 (ADOS‐2*** *):* a semi‐structured assessment allowing observations of social and communication behaviours relevant to the diagnosis of autism. We used module four of ADOS‐2 (designed for adolescents and adults with fluent speech) (Hus & Lord, 2014).

#### Online questionnaires

‐ ***Weiss Functional Impairment Rating Scale (WFIRS):*** a self‐report scale examining adult ADHD‐related impairment with 70 items covering seven domains of dysfunction: family relations, work adjustments, school performance, life skills, self‐concept, social functioning, and risk‐taking. It also provides two metrics of overall impairment: the total mean (mean impairment score from all items) and domain mean (mean domain scores, not weighted by the number of items in domain) (Canu et al., 2016).
‐ ***World Health Organization Quality of Life‐BREF (QoL)***: a self‐report questionnaire to assess the quality of life in four domains: physical health, psychological, social relationships and environmental (The WHOQOL Group, 1998). See Supporting Information for more details.

### Continuous performance task (CPT‐OX)

Participants were presented with the flanker version of the CPT‐OX (McLoughlin et al., 2010) consisting of a black letter array: a centre letter flanked on each side by distractor letters, presented in four identical blocks of 100 letter arrays each. Participants were instructed to ignore the distractor letters and attend only to the centre letter. Target centre letters ‘X’ and ‘O’ are flanked by the incompatible letter ‘O’ or ‘X’ and distractor letters are flanked by either ‘X’ or ‘O’. The 80 cues (XOX) initiated 40 cue‐target (go event) (XOX‐OXO) and 40 cue non‐target (no‐go event) sequences (XOX‐XDX). Participants were instructed to respond only to cue‐target sequences (XOX‐OXO) by pressing a button as quickly as possible with the index finger of their preferred hand. Letter arrays were presented briefly: (150 ms) every 1.65s in a pseudo‐random sequence. Task duration was 11 min.

### EEG recordings, pre‐processing and analysis

EEG was recorded with a mobile wireless 64‐channel (10‐10 montage) system (Cognionics, San Diego, CA; Ag/AgCl electrodes, sampling rate 2000 Hz). EEGLAB (Delorme & Makeig, 2004) and custom written MATLAB scripts (Mathworks, Natick, Massachusetts) were used for pre‐processing and analysis.

EEG data were downsampled to 256 Hz, bad EEG channels/time intervals were removed (see Supporting Information for details) and average reference was applied. Independent component analysis (ICA) was applied to each individual recording using Adaptive Mixture ICA (AMICA) (Palmer et al., 2011) with the EEGLAB nsgportal plug‐in on the Neuroscience Gateway (Martínez‐Cancino et al., 2021). Based on previous literature, ICA weights were calculated using 1–30 Hz filtered data and then the applied to 0.1–30 Hz filtered data (Winkler et al., 2015). This extra step ensures a high quality ICA decomposition while maintaining lower frequency ERP components of interest. Equivalent current dipoles were calculated for each IC component using the dipfit function from EEGLAB with a template four‐layer boundary element method head model (Oostendorp & van Oosterom, 1989). This function first performs dipole scanning on a coarse 3‐D grid to determine a starting position and afterwards uses a non‐linear optimization algorithm to find the exact dipole position for each component. We have used this function with its default parameters as implemented in EEGLAB (Localizing Independent Components using DIPFIT2, n.d.). We used the Eyecatch algorithm for detection and removal of ocular artefacts (Bigdely‐Shamlo, Kreutz‐Delgado, et al., 2013). Subsequently, continuous data were epoched −500−1650 ms at cue and no‐go events, baseline corrected, and bad epochs exceeding an amplitude threshold (±150 *μ*V) were removed. An EEGLAB study was then created from these epochs and dipoles located outside the brain or with higher than 15% residual variance were rejected.

ICA components were analysed using the Measure Projection Analysis (MPA) toolbox (Bigdely‐Shamlo, Mullen, et al., 2013). Signals measured at scalp EEG channels reflect a superposition of signals from many cortical sources, with weights of this summation depending on volume conduction. Source domains can be used to disentangle the scalp EEG and source‐based signals. ICA derived source measures have been shown to share more genetic variance with behaviour (including ADHD diagnosis) than channel‐based EEG measures (McLoughlin, Palmer, et al., 2014); thus both channel and source based signals were analysed. As the channel ERP represents a combination of potentials resulting from the volume conduction inherent in EEG and the ICA component ERP is an extracted potential optimized to be independent of other potential responses measured at the same electrode(s), the potential of the ICA components may be much lower amplitude than the raw channel potential. Thus, the ICA potential may have a different pattern from that in the larger composite channel potential as can be seen in the no‐go condition, especially around the time of P3.

MPA is a principled method used for clustering of ICA sources and projection of potentials to cortical domains and has been successfully used in analysis of the EEG data from the CPT task in McLoughlin et al. (2018). MPA involves finding voxels that are consistent across nearby source locations for given dynamic measures in a template brain space. In this study we used the IC time courses and dipoles as these measures. MPA computes local‐mean EEG measure values for this voxel subspace using a statistical model of source localization error and between‐subject anatomical variation. The last step is a type of clustering to find spatial domains exhibiting distinguishable measure features and provides 3‐D maps plus statistical significance estimates for each EEG measure of interest (Bigdely‐Shamlo, Mullen, et al., 2013). In line with previous work, we used a significance level of *p* = 0.01 and maximum domain exemplar correlation of 0.7 as the MPA parameters (McLoughlin et al., 2018).

### Selection of ERP measures and exploratory factor analysis (EFA)

**FIGURE 1:**
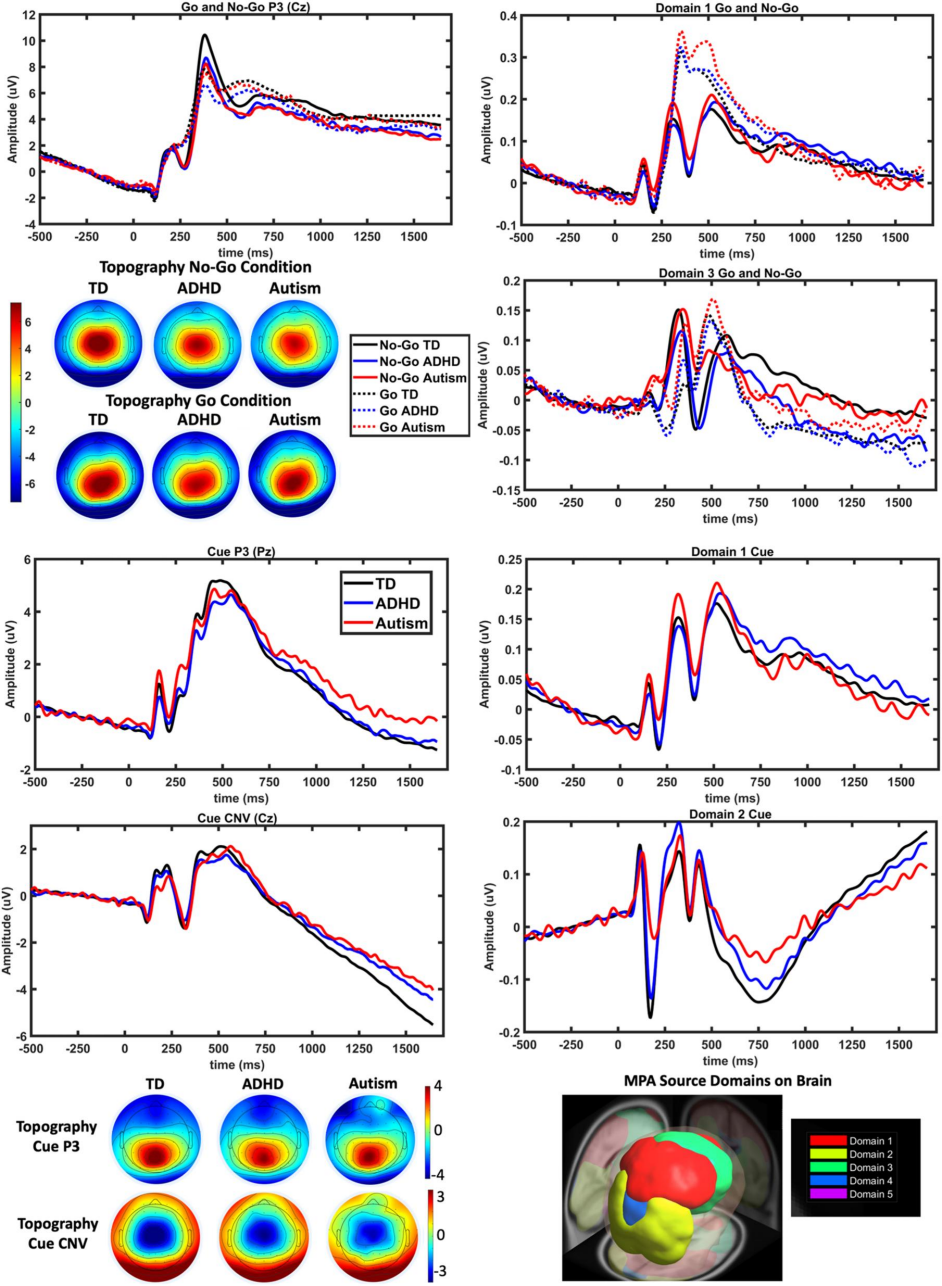
Channel‐based ERPs (left column) and MPA source domain signals (right column) for TD, ADHD, and autism groups. The peak (go, no‐go and cue P3) or average (CNV) topographies are shown. Domains 1 and 3 are shown for go and no‐go events, domains 1 and 2 for the cue events. ADHD, attention‐deficit/hyperactivity disorder; CNV, contingent negative variation; ERP, event‐related potential; MPA, Measure Projection Analysis; TD, typically developed

**FIGURE 2:**
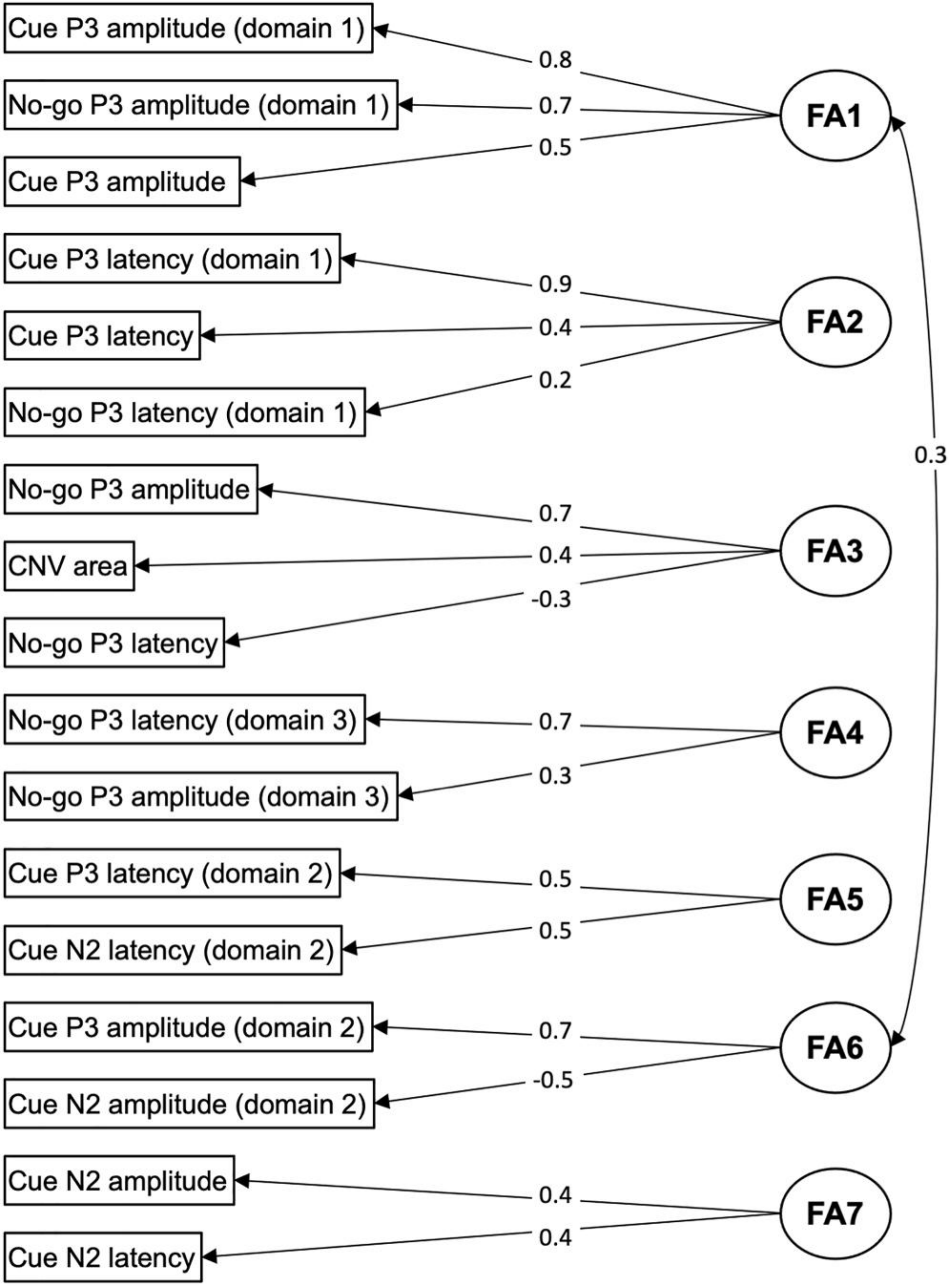
The EFA diagram showing the ERP variables with loadings higher than 0.2. MPA domain is indicated in parentheses. Specific areas corresponding to these domains are shown in Figure 1. EFA, exploratory factor analysis; ERP, event‐related potential; MPA, Measure Projection Analysis

Based on previous literature (e.g. (McLoughlin et al., 2010)) and the group level signals, ERP measures related to cue and no‐go events were determined (Figure 1A,B). Peak amplitude and latencies were calculated for all ERPs except for the CNV (area under the curve). The ERP components were channel‐based: cue CNV (1300–1650 ms, Cz), no‐go P3 (234–441 ms, Cz), cue P3 (355–550 ms, Pz), cue N2 (188–242 ms, Pz), and source‐based: no‐go P3 (456–589 ms, domain 1), no‐go P3 (283–381 ms, domain 3), cue P3 (424–655 ms, domain 1), cue P3 (252–350 ms, domain 2), cue N2 (142–217 ms, domain 2). Time intervals for ERPs were based on the latency of prominent peaks on grand averaged data, calculated by averaging over all subjects regardless of their group. An interval around these peaks was selected and individual participant signals were plotted to ensure that the selected interval covered individual peaks without interfering with other peaks of interest. Using the Psych package in R (Revelle, 2017), exploratory factor analysis (EFA) was applied to the 17 ERP measures to determine the main factors for further analysis. As determined by the parallel analysis method, seven factors were retained (Horn, 1965). Though there was a tendency for the factors to represent amplitude or latency measures separately, this was not always the case. Furthermore, there was no general simple division of ERP measures into channel versus source measures, or proactive/inhibitory control measures (Figure 2).

### Twin modelling

Twin modelling is based on fitting a genetic model that explains differential correlations observed in twin data by considering the MZ twins share 100% and DZ twins share 50% of their genetic influences while MZ and DZ twins equally share environmental factors (Neale & Maes, 2004). OpenMx was used for twin modelling (Boker et al., 2011). Twin correlations and variance decomposition models for MZ and DZ data were calculated with liability threshold models used for ADHD (binarised from DIVA‐2) and autism (binarised from ADOS‐2). Liability threshold models are designed to handle dichotomous variables such as the existence of a disorder and assumes the risk to a disorder as normally distributed and an individual will have the disorder when a certain threshold is exceeded (Rijsdijk & Sham, 2002). Trait variance and covariance were estimated into standardised genetic variance (a^2^), common environment variance (c^2^), and unique environment variance and measurement error (e^2^). Considering most genetic variance is additive (Hill et al., 2008) and the lack of power in our sample to estimate additive (a^2^) and dominance (d^2^) genetics separately, we only used ACE models which will mean dominance genetic variance will go in a^2^ and therefore a^2^ reflects broad sense heritability.

Since the sample was selected on affection status of either ADHD or autism, some corrections are needed within the standard twin model. Correction for sample selection involves adjustments of the fit function for parts of the distribution that are unobserved. However, when using raw maximum likelihood estimation methods, a simpler fix can be obtained by including the selection variable(s) in the analyses. If these are diagnostic measures and only certain extreme groups are included (e.g., concordant or discordant affected twins), then in addition we also need to fix the model parameters. We fixed the thresholds, set to the population prevalence of 4% for ADHD (Fayyad et al., 2007) and 1% for autism (Colvert et al., 2015). We fixed heritability parameters (and associated twin correlations), set to population estimates: *a* ^2^ = 0.76, *c* ^2^ = 0, *e* ^2^ = 0.24, and rMZ = 0.76, rDZ = 0.38, using the same estimates for ADHD and autism as justified by previous studies (Colvert et al., 2015; Faraone et al., 2005; McLoughlin, Palmer, et al., 2014). The variance components (A, C, E) of the additional variables in the model are free to be estimated and so are their genetic (Ra) and environmental correlations (Rc and Re) with ADHD and autism. From these estimates the phenotypic correlations Rph‐a, Rph‐c, Rph‐e which adds up to Rph‐total were derived. Likelihood‐based asymmetric 95% confidence intervals (CIs) were estimated for all parameters in OpenMx. Three different models were fitted on raw data of the whole sample, including typically developed (TD) individuals (see Supporting Information and Figure S1).

ERP and WFIRS/QoL measures were preselected for twin modelling using multilevel mixed effects models to test for phenotypic (independent of twin relatedness) relationships. In these models, age and sex were accounted for as covariates and a random intercept was used to control for twin relatedness (Malone et al., 2014). Only the variables related with each other with a trend of *p* < 0.1 in the phenotypic analysis were included in the genetic models. Throughout this study we will only refer to the a^2^, c^2^ and e^2^ estimates in the model selected on ADHD because the estimates vary little whether they were examined in the model with ADHD or autism.

## RESULTS

In this study we have included the 16 participants who has both ADHD and autism to both groups. The results when comorbid cases are excluded (Tables S2 and S3), and interpretations can be found in the Supporting Information.

### Are ADHD and autism associated with quality of life and functional impairment in young adults? What are the contributions of genetics to these phenotypic correlations?

**FIGURE 3:**
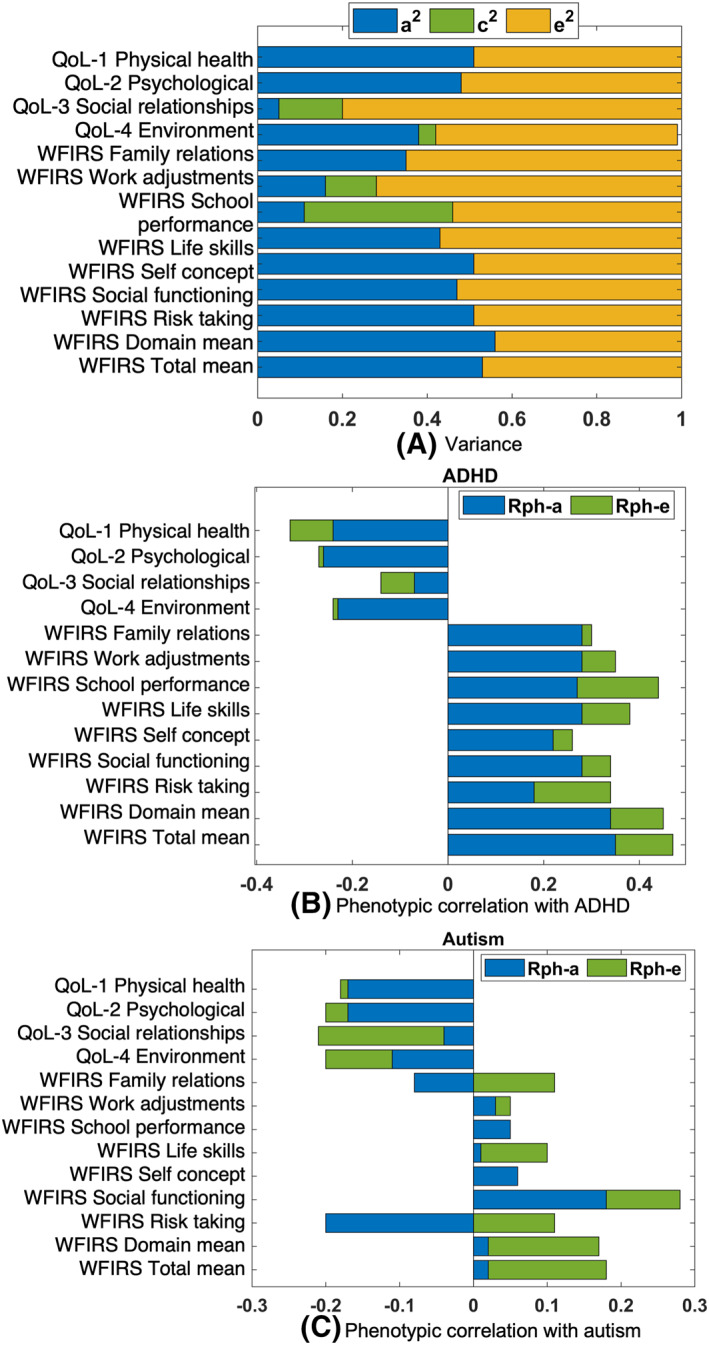
(A) The variances for QoL and WFIRS measures estimated by twin analysis. The figure shows the estimates from the two separate bivariate models (with ADHD and autism) are similar (Table 1). (B), (C) The phenotypic correlations estimated between QoL and WFIRS measures and ADHD or autism. 95% CIs are provided in Table 1. ADHD, attention‐deficit/hyperactivity disorder; WFIRS, Weiss Functional Impairment Rating Scale

Genetics significantly contributed both to QoL and WFIRS (a^2^ column, Figure 3 and Table 1). All four QoL domains (physical health, psychological, social relationships, and environment) had significant and negative phenotypic correlations with ADHD (−0.33, −0.28, −0.14 and −0.24, respectively) and autism (−0.18, −0.20, −0.22 and −0.20, respectively) (Table 1). Shared genetics (Rph‐a) made a significant contribution to the phenotypic correlation between ADHD and physical health (73%), psychological (93%) and environment (96%) QoL. In line with this, significant genetic correlations (Ra) were found between ADHD and physical health (−0.39), psychological (−0.44) and environment (−0.43) QoL as well as between autism and physical health (−0.28) QoL (Table 1).

**TABLE 1:** Findings of the bivariate twin model to investigate the heritability of QoL and WFIRS and the association of these measures to ADHD and autism

Measures	Condition	rMZ	rDz	*a* ^2^	*c* ^2^	*e* ^2^	Rph‐total	Rph‐a	Rph‐e	Ra
QoL‐1 Physical health	ADHD	**0.51**	**0.25**	**0.51**	*0.00*	**0.49**	**−0.33**	**−0.24**	−0.09	**−0.39**
		0.37, 0.63	0.07, 0.41	0.13, 0.62	*0.00*, *0.30*	0.38, 0.63	−0.43, −0.23	−0.36, −0.12	−0.18, 0.01	−0.83, −0.19
	Autism	**0.52**	**0.27**	**0.50**	*0.03*	**0.48**	**−0.18**	−0.17	−0.01	**−0.28**
		0.38, 0.63	0.09, 0.43	0.09, 0.63	*0.00*, *0.36*	0.37, 0.62	−0.30, −0.05	−0.34, 0.00	−0.15, 0.14	−0.79, −0.38
QoL‐2 Psychological	ADHD	**0.48**	**0.23**	**0.48**	*0.00*	**0.52**	**−0.28**	**−0.26**	−0.01	**−0.44**
		0.32, 0.60	0.04, 0.40	0.07, 0.60	*0.00*, *0.35*	0.40, 0.68	−0.37, −0.17	−0.38, −0.14	−0.11, 0.08	−1.00, −0.23
	Autism	**0.50**	**0.23**	**0.50**	*0.00*	**0.50**	**−0.20**	−0.17	−0.03	−0.27
		0.35, 0.62	0.03, 0.41	0.09, 0.61	*0.00*, *0.33*	0.39, 0.64	−0.32, −0.07	−0.34, 0.01	−0.19, 0.13	−0.57, 0.01
QoL‐3 Social relationships	ADHD	**0.20**	0.19	*0.05*	*0.15*	**0.80**	**−0.14**	−0.07	−0.07	−0.36
		0.02, 0.37	0.00, 0.36	*0.00*, *0.37*	*0.00*, *0.31*	0.63, 0.94	−0.24, −0.03	−0.20, 0.06	−0.18, 0.05	−1.00, 1.00
	Autism	**0.21**	0.19	*0.05*	*0.16*	**0.79**	**−0.22**	−0.04	−0.17	−0.22
		0.03, 0.38	0.00, 0.36	*0.00*, *0.38*	*0.00*, *0.32*	0.62, 0.93	−0.33, −0.09	−0.23, 0.13	−0.34, 0.02	−1.00, 1.00
QoL‐4 environment	ADHD	**0.43**	**0.25**	**0.38**	*0.04*	**0.57**	**−0.24**	**−0.23**	−0.01	**−0.43**
		0.28, 0.55	0.04, 0.42	0.03, 0.55	*0.00*, *0.38*	0.45, 0.72	−0.34, −0.14	−0.35, −0.11	−0.11, 0.09	−1.00, −0.19
	Autism	**0.44**	**0.27**	*0.33*	*0.12*	**0.56**	**−0.20**	−0.11	−0.09	−0.22
		0.30, 0.56	0.06, 0.44	*0.00*, *0.56*	*0.00*, *0.44*	0.44, 0.70	−0.32, −0.08	−0.29, 0.06	−0.25, 0.08	−1.00, 1.00
WFIRS family relations	ADHD	**0.40**	0.06	**0.35**	*0.00*	**0.65**	**0.30**	**0.28**	0.02	**0.55**
		0.22, 0.55	−0.12, 0.24	0.08, 0.50	*0.00*, *0.20*	0.50, 0.82	0.20, 0.40	0.16, 0.40	−0.10, 0.13	0.30, 1.00
	Autism	**0.43**	0.10	**0.39**	*0.00*	**0.61**	0.04	−0.08	0.11	−0.14
		0.26, 0.57	−0.09, 0.28	0.36, 0.54	*0.00*, *0.22*	0.46, 0.77	−0.09, 0.17	−0.24, 0.09	−0.04, 0.25	−0.47, 0.17
WFIRS work adjustments	ADHD	**0.27**	0.18	**0.16**	*0.12*	**0.73**	**0.35**	**0.28**	0.07	**0.80**
		0.07, 0.45	−0.08, 0.41	0.03, 0.45	*0.00*, *0.32*	0.55, 0.90	0.24, 0.45	0.14, 0.40	−0.05, 0.19	0.31, 1.00
	Autism	**0.31**	0.24	*0.15*	*0.16*	**0.69**	0.06	0.03	0.02	0.10
		0.10, 0.48	−0.03, 0.45	*0.00*, *0.48*	*0.00*, *0.42*	0.52, 0.88	−0.09, 0.21	−0.16, 0.23	−0.18, 0.21	−1.00, 1.00
WFIRS school performance	ADHD	**0.45**	**0.42**	**0.11**	*0.35*	**0.55**	**0.43**	**0.27**	**0.17**	**0.94**
		0.14, 0.65	0.14, 0.61	0.02, 0.64	*0.00*, *0.52*	0.35, 0.75	0.31, 0.54	0.12, 0.40	0.05, 0.28	0.87, 1.00
	Autism	**0.53**	**0.45**	*0.14*	*0.37*	**0.48**	0.05	0.05	0.00	0.15
		0.24, 0.71	0.16, 0.64	*0.00*, *0.68*	*0.00*, *0.62*	0.30, 0.71	−0.13, 0.22	−0.16, 0.26	−0.22, 0.19	−1.00, 1.00
WFIRS life skills	ADHD	**0.45**	0.15	**0.43**	*0.00*	**0.57**	**0.38**	**0.28**	0.10	**0.49**
		0.29, 0.58	−0.03, 0.32	0.10, 0.56	*0.00*, *0.25*	0.44, 0.73	0.28, 0.47	0.16, 0.40	0.00, 0.20	0.28, 1.00
	Autism	**0.48**	0.17	**0.46**	*0.00*	**0.54**	0.11	0.01	0.09	0.02
		0.32, 0.61	−0.02, 0.35	0.13, 0.59	*0.00*, *0.26*	0.41, 0.69	−0.03, 0.23	−0.15, 0.17	−0.05, 0.23	−0.26, 0.29
WFIRS self‐concept	ADHD	**0.51**	**0.24**	**0.51**	*0.00*	**0.49**	**0.26**	**0.22**	0.04	**0.36**
		0.36, 0.63	0.05, 0.40	0.11, 0.62	*0.00*, *0.32*	0.38, 0.64	0.16, 0.36	0.10, 0.34	−0.06, 0.14	0.15, 0.85
	Autism	**0.53**	**0.25**	**0.52**	*0.00*	**0.48**	0.05	0.06	0.00	0.09
		0.38, 0.64	0.06, 0.41	0.12, 0.64	*0.00*, *0.33*	0.36, 0.61	−0.08, 0.19	−0.12, 0.23	−0.16, 0.15	−0.20, 0.36
WFIRS social functioning	ADHD	**0.53**	0.05	**0.47**	*0.00*	**0.53**	**0.34**	**0.28**	0.06	**0.47**
		0.38, 0.65	−0.14, 0.23	0.25, 0.61	*0.00*, *0.14*	0.39, 0.69	0.24, 0.44	0.15,0.40	−0.04, 0.17	0.26, 0.71
	Autism	**0.56**	0.04	**0.50**	*0.00*	**0.50**	**0.28**	**0.18**	0.10	**0.29**
		0.41, 0.67	−0.16, 0.23	0.30, 0.63	*0.00*, *0.13*	0.37, 0.66	0.15, 0.40	0.01, 0.34	−0.06, 0.24	0.07, 0.55
WFIRS risk taking	ADHD	**0.55**	0.06	**0.51**	*0.00*	**0.49**	**0.34**	**0.18**	**0.16**	**0.30**
		0.41, 0.66	−0.14, 0.25	0.29, 0.63	*0.00*, *0.16*	0.37, 0.64	0.24, 0.44	0.07, 0.30	0.06, 0.24	0.11, 0.49
	Autism	**0.57**	0.02	**0.53**	*0.00*	**0.47**	−0.09	**−0.20**	0.11	**−0.32**
		0.44, 0.68	−0.19, 0.22	0.34, 0.65	*0.00*, *0.13*	0.35, 0.63	−0.22, 0.04	−0.37, −0.02	−0.07, 0.26	−0.60, −0.03
WFIRS domain mean	ADHD	**0.58**	0.17	**0.56**	*0.00*	**0.44**	**0.45**	**0.34**	**0.11**	**0.52**
		0.44, 0.68	−0.02, 0.34	0.30, 0.67	*0.00*, *0.19*	0.33, 0.58	0.35, 0.54	0.22, 0.45	0.01, 0.20	0.34, 0.76
	Autism	**0.61**	0.19	**0.59**	*0.00*	**0.41**	**0.17**	0.02	0.15	0.03
		0.48, 0.71	−0.01, 0.37	0.35, 0.69	*0.00*, *0.20*	0.31, 0.54	0.04, 0.30	−0.14, 0.19	0.00, 0.27	−0.21, 0.28
WFIRS total mean	ADHD	**0.56**	0.15	**0.53**	*0.00*	**0.47**	**0.48**	**0.35**	**0.12**	**0.55**
		0.42, 0.67	−0.03, 0.33	0.29, 0.65	*0.00*, *0.18*	0.35, 0.61	0.38, 0.56	0.23, 0.47	0.02, 0.22	0.37, 0.81
	Autism	**0.60**	0.17	**0.58**	*0.00*	**0.42**	**0.18**	0.02	**0.16**	0.03
		0.46, 0.70	−0.02, 0.35	0.34, 0.68	*0.00*, *0.19*	0.32, 0.56	0.04, 0.31	−0.14, 0.19	0.01, 0.28	−0.22, 0.28

*Note*: For each questionnaire two bivariate models were used, one with ADHD and one with autism, and they are both included in the tables; **rMz:** MZ twin correlations; **rDZ:** DZ twin correlations. 95% confidence intervals (CIs) are included under each estimate and significant estimate are written in bold. Point estimates shown in italic reflect the cases where the confidence interval was close to zero (showing 0.00 due to rounding, though not overlapping with zero), in some cases this might be due to limited statistical power in the model to identify the genetic and common environment contribution separately. These results should be interpreted with caution.

We found significant phenotypic correlations between each WFIRS domain and ADHD (Table 1). The contribution of genetics to the phenotypic correlations was significant across all domains: family relations (93%), work adjustments (80%), school performance (63%), life skills (74%), self‐concept (85%), social functioning (82%), risk‐taking (53%), domain mean (76%), total mean (73%) with significant moderate to high genetic correlations emerging, ranging from 0.30 for risk taking to 0.94 for school performance (Figure 3 and Table 1).

Social functioning was the only WFIRS specific domain with a significant phenotypic correlation with autism (0.28). The genetic contribution to this correlation was also significant (64%). While the total phenotypic correlation between autism and risk‐taking was not statistically significant, the genetic contribution was statistically significant (−0.20) (Table 1). This is line with a significant genetic correlation between autism and risk taking (−0.32), in addition to significant genetic overlap between autism and social functioning (0.29) (Table 1). Autism was associated with mean functional impairment (domain mean (0.17); total mean (0.18)) (Table 1).

### Are ERP measures from the CPT‐OX associated with ADHD and autism in young adults?

**FIGURE 4:**
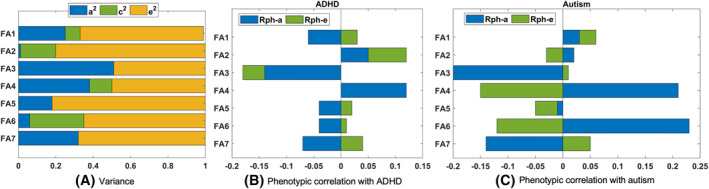
(A) The variances for ERP factors estimated by twin analysis. Figure showing the estimates from the bivariate model with ADHD (B), (C) The phenotypic correlations estimated between ERP factors and ADHD or autism. ADHD, attention‐deficit/hyperactivity disorder; ERP, event‐related potential

Grand average channel‐based ERP waveforms and scalp topographic maps, brain domains calculated with MPA and the respective source‐based grand average ERP waveforms are shown in Figure 1. The measures contributing to each ERP factor are shown in Figure 2. ERP factors were heritable despite in many cases the lower estimate of the confidence intervals being close to zero (indicated with italics in Table 2). The highest heritability estimates were for FA3 (0.51), FA4 (0.38) and FA7 (0.32). Common environment (*c* ^2^) contributions were highest for FA6 (0.29) and FA2 (0.19) (Figure 4 and Table 2). FA3 showed significant negative phenotypic correlations with both ADHD (−0.18) and autism (−0.19) (Figure 4 and Table 2). The genetic contribution to these relationships were significant: 78% (−0.14) for ADHD and −0.20 for autism (please note that when there are different signs for genetic and (unique) environmental correlations, as the case here for autism, they cannot be indicated as percentages). In agreement with this, the genetic correlations were significant: ADHD (−0.23) and autism (−0.32). Distinct relationships emerged for other ERP measures and ADHD and autism. FA2 and FA4 had significant phenotypic correlations with ADHD (both 0.12); however, genetic and environment contributions were not significant. The point estimate of the genetic contribution for FA4 was 100% but failed significance by small margin (Table 2 and Figure 4). While the phenotypic correlations between autism and FA4 and FA6 were not significant, the genetic contributions and correlations were statistically significant at 0.21 (Ra = 0.38) and 0.23 (Ra = 1.00), respectively.

**TABLE 2:** Findings of the bivariate twin model to investigate the heritability of seven ERP factors and the association of these factors to ADHD and autism

ERP factors	Condition	rMZ	rDz	*a* ^2^	*c* ^2^	*e* ^2^	Rph‐total	Rph‐a	Rph‐e	Ra
FA1	ADHD	**0.34**	**0.21**	*0.25*	*0.08*	**0.66**	−0.03	−0.06	0.03	−0.14
		0.15, 0.49	0.04, 0.36	*0.00*, *0.49*	*0.00*, *0.36*	0.51, 0.84	−0.13, 0.08	−0.19, 0.07	−0.08, 0.14	−1.00, 1.00
	Autism	**0.34**	**0.21**	*0.26*	*0.08*	**0.66**	0.06	0.03	0.03	0.08
		0.15, 0.49	0.04, 0.36	*0.00*, *0.49*	*0.00*, *0.36*	0.51, 0.84	−0.06, 0.19	−0.13, 0.20	−0.13, 0.19	−1.00, 1.00
FA2	ADHD	**0.20**	**0.19**	*0.01*	*0.19*	**0.80**	**0.12**	0.05	0.07	0.46
		0.01, 0.37	0.02, 0.35	*0.00*, *0.37*	*0.00*, *0.31*	0.63, 0.93	0.02, 0.22	−0.08, 0.17	−0.03, 0.18	−1.00, 1.00
	Autism	*0.20*	**0.19**	*0.02*	*0.18*	**0.80**	−0.01	0.02	−0.03	0.15
		*0.00*, *0.37*	0.02, 0.35	*0.00*, *0.37*	*0.00*, *0.32*	0.63, 0.93	−0.13, 0.12	−0.15, 0.19	−0.20, 0.15	−1.00, 1.00
FA3	ADHD	**0.52**	**0.22**	**0.51**	*0.00*	**0.49**	**−0.18**	**−0.14**	−0.04	**−0.23**
		0.36, 0.64	0.06, 0.37	0.17, 0.63	*0.00*, *0.25*	0.37, 0.64	−0.28, −0.08	−0.27, −0.02	−0.14, 0.06	−0.49, −0.02
	Autism	**0.53**	**0.23**	**0.52**	*0.00*	**0.48**	**−0.19**	**−0.20**	0.01	**−0.32**
		0.37, 0.64	0.07, 0.37	0.17, 0.63	*0.00*, *0.26*	0.37, 0.63	−0.31, −0.07	−0.35, −0.04	−0.13, 0.15	−0.57, −0.16
FA4	ADHD	**0.50**	**0.31**	*0.38*	*0.12*	**0.50**	**0.12**	0.12	0.00	0.22
		0.33, 0.63	0.16, 0.45	*0.00*, *0.63*	*0.00*, *0.42*	0.37, 0.67	0.01, 0.22	−0.01, 0.24	−0.10, 0.10	−0.03, 1.00
	Autism	**0.51**	**0.31**	**0.39**	*0.12*	**0.49**	0.05	**0.21**	*−0.15*	**0.38**
		0.33, 0.64	0.16, 0.44	0.02, 0.64	*0.00*, *0.41*	0.36, 0.66	−0.07, 0.18	0.04, 0.36	*−0.29*, *0.00*	0.07, 1.00
FA5	ADHD	0.21	0.06	*0.18*	*0.00*	**0.82**	−0.02	−0.04	0.02	−0.11
		−0.01, 0.39	−0.11, 0.21	*0.00*, *0.35*	*0.00*, *0.22*	0.65, 1.00	−0.12, 0.08	−0.17, 0.09	−0.10, 0.15	−1.00, 1.00
	Autism	0.21	0.07	*0.18*	*0.00*	**0.82**	−0.05	−0.01	−0.04	−0.03
		−0.01, 0.39	−0.10, 0.22	*0.00*, *0.35*	*0.00*, *0.23*	0.65, 1.00	−0.18, 0.08	−0.19, 0.16	−0.22, 0.15	−1.00, 1.00
FA6	ADHD	**0.35**	**0.32**	*0.06*	*0.29*	**0.65**	−0.03	−0.04	0.01	−0.17
		0.14, 0.51	0.17, 0.45	*0.00*, *0.49*	*0.00*, *0.44*	0.49, 0.79	−0.13, 0.07	−0.16, 0.09	−0.10, 0.11	−1.00, 1.00
	Autism	**0.32**	**0.32**	*0.07*	*0.27*	**0.66**	0.11	**0.23**	−0.12	**1.00**
		0.10, 0.50	0.17, 0.45	*0.00*, *0.46*	*0.00*, *0.40*	0.55, 0.79	−0.02, 0.23	0.05, 0.38	−0.27, 0.06	0.16, 1.00
FA7	ADHD	**0.32**	0.16	*0.32*	*0.00*	**0.68**	−0.03	−0.07	0.04	−0.14
		0.12, 0.49	−0.01, 0.31	*0.00*, *0.47*	*0.00*, *0.31*	0.53, 0.85	−0.13, 0.08	−0.20, 0.06	−0.08, 0.16	−1.00, 1.00
	Autism	**0.33**	0.16	*0.33*	*0.00*	**0.67**	−0.09	−0.14	0.05	−0.28
		0.13, 0.49	0.00, 0.31	*0.00*, *0.47*	*0.00*, *0.30*	0.53, 0.84	−0.21, 0.04	−0.30, 0.03	−0.13, 0.22	−1.00, 0.07

*Note*: For each ERP factor two bivariate models were used, one with ADHD and one with autism, and they are both included in the tables; rMz: MZ twin correlations; rDZ: DZ twin correlations. 95% confidence intervals (CIs) are included under each estimate and significant estimate are written in bold. Point estimates shown in italic reflect the cases where the confidence interval was close to zero (showing 0.00 due to rounding, though not overlapping with zero), in some cases this might be due to limited statistical power in the model to identify the genetic and common environment contribution separately. These results should be interpreted with caution.

### Are ERP measures from the CPT‐OX associated with QoL or functional impairment?

**FIGURE 5:**
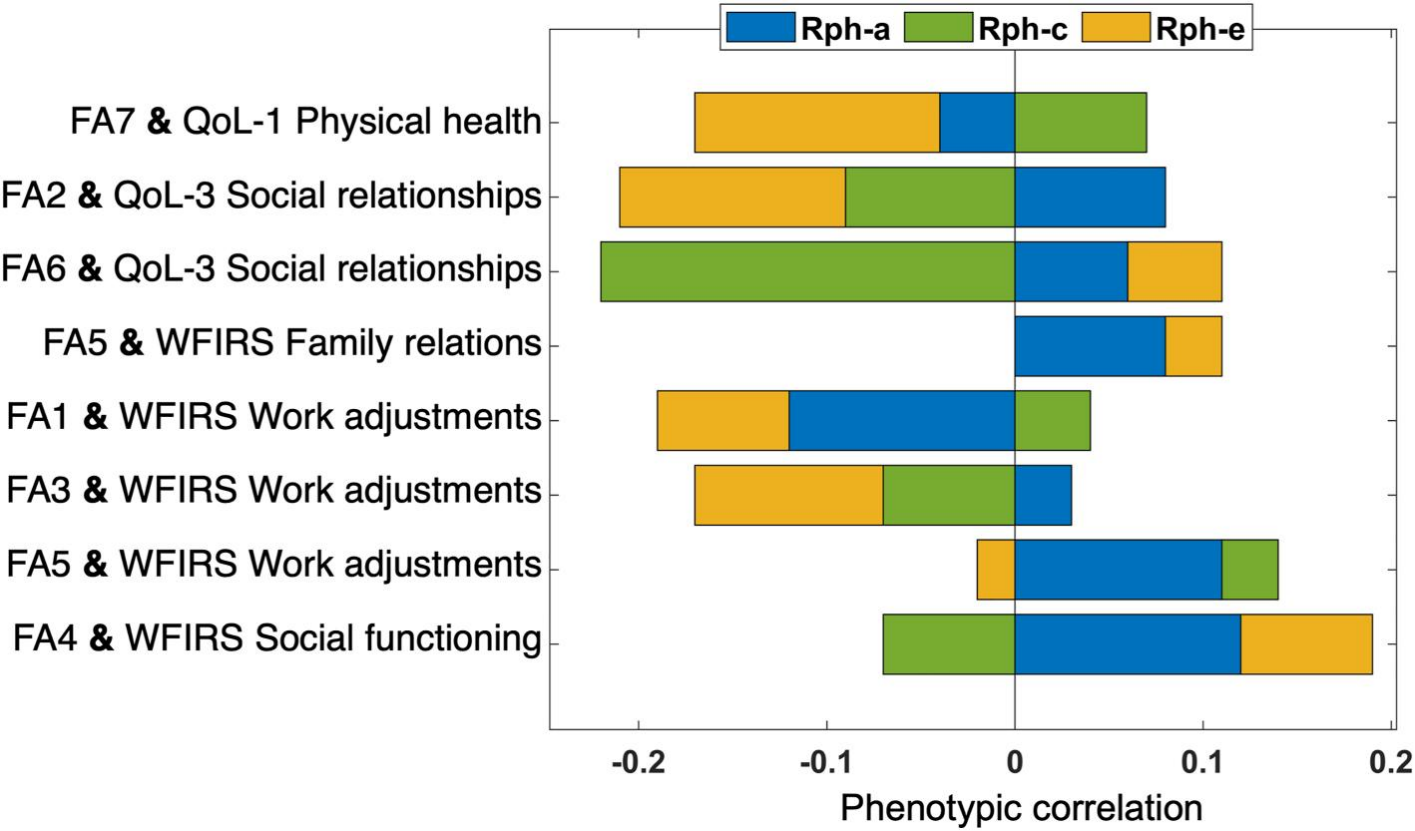
The phenotypic correlations estimated between ERP factors and QoL/WFIRS measures. The figure shows the estimates from the trivariate model with ADHD. ADHD, attention‐deficit/hyperactivity disorder; ERP, event‐related potential

Work adjustment (WFIRS) was phenotypically associated with a broad representation of ERP factors: FA1 (−0.14), FA3 (−0.13) and FA5 (0.12); however, the contribution of genetics to these relationships were not statistically significant. Social relationships (QoL) showed significant negative phenotypic correlations with FA2 (−0.12) and FA6 (−0.10), with significant common environment contributions for FA6 at −0.22 (Rc = 1.00). Social functioning (WFIRS) showed a different pattern of results: a small yet significant relationship emerged between FA4 and social functioning (0.12) (Figure 5 and Table 3). Physical health significantly correlated with FA7 (−0.11) but with only a significant unique environment (and error) contribution (−0.13, Figure 5 and Table 3). A significant phenotypic correlation emerged between family relations and FA5 (0.11) (Figure 5 and Table 3). Path estimates (a_32_) between social QoL and FA2 and FA6 as well as work adjustments and FA1 and FA5 suggested considerable association between ERP factors and these WFIRS and QoL measures independent of ADHD and autism (Table S4).

**TABLE 3:** Findings of the trivariate twin model with WFIRS or QoL, ERP factors and ADHD or autism

Variables	Condition	Rph‐total	Rph‐a	Rph‐c	Rph‐e	Ra	Rc
FA7 QoL‐1 physical health	ADHD	**−0.11**	−0.04	0.07	**−0.13**	−0.14	1.00
		−0.20, −0.02	−0.23, 0.19	−0.11, 0.19	−0.25, −0.02	−1.00, 1.00	−1.00, 1.00
	Autism	**−0.12**	−0.05	0.06	**−0.13**	−0.13	1.00
		−0.21, −0.02	−0.23, 0.22	−0.14, 0.19	−0.24, −0.03	−0.96, 1.00	−1.00, 1.00
FA2 QoL‐3 social relationships	ADHD	**−0.12**	0.08	−0.09	−0.12	0.90	−0.69
		−0.22, −0.03	−0.18, 0.18	−0.18, 0.12	−0.24, 0.01	−1.00, 1.00	−1.00, 1.00
	Autism	**−0.14**	0.11	−0.11	**−0.14**	0.98	−0.92
		−0.23, −0.04	−0.17, 0.19	−0.19, 0.12	−0.25, −0.01	−1.00, 1.00	−1.00, 1.00
FA6 QoL‐3 social relationships	ADHD	**−0.11**	0.06	**−0.22**	0.05	0.99	**−1.00**
		−0.20, −0.02	−0.11, 0.14	−0.29, −0.09	−0.06, 0.16	−1.00, 1.00	−1.00, −0.42
	Autism	**−0.10**	0.05	**−0.20**	0.05	0.58	**−1.00**
		−0.19, −0.01	−0.11, 0.14	−0.27, −0.06	−0.06, 0.16	−1.00, 1.00	−1.00, −0.93
FA5 WFIRS family relations	ADHD	**0.11**	0.08	0.00	0.03	0.31	0.66
		0.02, 0.20	−0.12, 0.22	−0.08, 0.14	−0.10, 0.16	−1.00, 1.00	−1.00, 1.00
	Autism	**0.11**	0.08	0.00	0.03	0.29	−0.60
		0.01, 0.20	−0.12, 0.25	−0.11, 0.13	−0.09, 0.16	−1.00, 1.00	−1.00, 1.00
FA1 WFIRS work adjustments	ADHD	**−0.14**	−0.12	0.04	−0.07	−0.63	0.36
		−0.24, −0.04	−0.28, 0.16	−0.18, 0.15	−0.22, 0.06	−1.00, 1.00	−1.00, 1.00
	Autism	**−0.14**	−0.09	0.00	−0.05	−0.49	−0.03
		−0.24, −0.04	−0.31, 0.19	−0.23, 0.16	−0.19, 0.09	−1.00, 1.00	−1.00, 1.00
FA3 WFIRS work adjustments	ADHD	**−0.13**	0.03	−0.07	−0.10	0.14	−1.00
		−0.23, −0.03	−0.21, 0.19	−0.18, 0.11	−0.21, 0.02	−0.82, 0.73	−1.00, 1.00
	Autism	**−0.14**	0.03	−0.08	−0.08	0.11	−1.00
		−0.24, −0.04	−0.24, 0.22	−0.23, 0.11	−0.20, 0.04	−1.00, 1.00	−1.00, 1.00
FA5 WFIRS work adjustments	ADHD	**0.12**	0.11	0.03	−0.02	0.62	1.00
		0.02, 0.21	−0.10, 0.26	−0.07, 0.20	−0.15, 0.12	−1.00, 1.00	−1.00, 1.00
	Autism	**0.11**	0.15	0.00	−0.03	1.00	−1.00
		0.01, 0.21	−0.12, 0.28	−0.10, 0.21	−0.16, 0.12	−1.00, 1.00	−1.00, 1.00
FA4 WFIRS social functioning	ADHD	**0.12**	0.12	−0.07	0.07	0.33	−1.00
		0.02,0.21	−0.10, 0.31	−0.20, 0.08	−0.03, 0.18	−0.49, 1.00	−1.00, 1.00
	Autism	**0.13**	0.11	−0.06	0.08	**0.30**	−1.00
		0.04, 0.23	−0.10, 0.31	−0.20, 0.09	−0.02, 0.19	0.30, 1.00	−1.00, 1.00

*Note*: ADHD and autism variables were only used to avoid bias due to using an enriched study sample. The phenotypic correlations estimated with the trivariate model between ADHD or autism and these ERP factors or questionnaires were very similar to the ones reported with bivariate models (Tables 1 and 2). **Rph‐total:** Total phenotypic correlation between the ERP factor and QoL or WFIRS. 95% confidence intervals (CIs) are included under each estimate and significant estimate are written in bold. Point estimates shown in italic reflect the cases where the confidence interval was close to zero (showing 0.00 due to rounding, though not overlapping with zero), in some cases this might be due to limited statistical power in the model to identify the genetic and common environment contribution separately. These results should be interpreted with caution.

## DISCUSSION

We examined the phenotypic, genetic, and environmental relationships between ADHD/autism, functional impairment (WFIRS), quality of life (QoL), and ERP measures (CPT‐OX task) in 283 young adult twin pairs. Significant phenotypic correlations emerged between both ADHD and autism and QoL in all four domains of physical health, psychological, social relationships, and environment. The association of ADHD and autism diagnosis with lower QoL agrees with previous studies in both autism (Capp et al., 2022; Mason et al., 2018) and ADHD (Quintero et al., 2019). Importantly, these findings indicate large shared genetic aetiology between these conditions and QoL, particularly for ADHD.

ADHD was associated with increased functional impairment across all seven domains of the WFIRS and overall impairment, in line with previous literature (Canu et al., 2016; Weiss et al., 2018). Novel findings emerged in the large and significant genetic overlap between ADHD and functional impairment across multiple domains (53%–93%). Autism was associated both phenotypically and genetically with impairment in social functioning consistent with central deficits in social communication in the condition (American Psychiatric Association, 2013). In accordance with previous findings (De Groot, 2020; South et al., 2014), autism also showed a significant relationship with decreased risk raking; though to date no other study has demonstrated our finding of genetic contributions to this overlap. It has been suggested that reduced risk‐taking in autism is related to decreased motivation to engage in social activities, which results in a lack of experience, which may in turn result in wariness for such activities (Chevallier et al., 2012; De Groot, 2020). Alternatively, it has been suggested that the perception of risk may be inherent to the lack of engagement in social activities in autism (De Groot, 2020).

In agreement with previous literature on the CPT‐OX and these conditions, FA3, representing the amplitude and latency of the channel measures of the no‐go P3 and the CNV, emerged as having the largest genetic and phenotypic correlations with both ADHD and autism, indicating impairments in proactive and inhibitory control (Cheung et al., 2016; McLoughlin et al., 2010; Rommel et al., 2017; Tye et al., 2014). This agrees with a large meta‐analysis indicating that two of the largest differences between individuals with ADHD and those without are the amplitude of the no‐go P3 and the CNV (Kaiser et al., 2020). Frontal source contributions to the no‐go P3 (FA4) showed a specific significant phenotypic correlation with ADHD. Furthermore, ADHD specifically correlated with longer latencies for cue P3 and no‐go P3 (FA2), consistent with slower processing in the disorder (Banaschewski et al., 2003; Kaiser et al., 2020; McLoughlin et al., 2010). Numerous experimental studies have related the no‐go P3 specifically to inhibitory control (Albrecht et al., 2013; Gajewski & Falkenstein, 2013; Liotti et al., 2005). The association with ADHD is identified independently of the P3 to ‘go’ stimuli in the CPT‐OX, which has not been found to be associated with the disorder (Albrecht et al., 2013; Doehnert et al., 2010; McLoughlin et al., 2010; Michelini et al., 2021). However, underlying processes common across conditions of the CPT‐OX may be of interest in future work using additional ERP or time‐frequency analysis.

Parietal‐occipital source contributions to both the P3 and N2 at cue events were specifically associated with autism (FA6). The negative loading for the amplitude of cue N2 and the positive loading for cue P3 amplitude at the parietal‐occipital source domain (Figure 2) indicate lower cue N2 and higher cue P3 amplitudes in autism. This is in agreement with previous findings in autistic young adults indicating impairment in orientation of initial attention (Wang et al., 2017). The enhanced cue P3 is consistent with evidence of increased P3 amplitude to predictive stimuli in autistic adults; interpreted as a resistance to uncertainty and thus over‐anticipation of stimuli (Thillay et al., 2016).

Despite observing significant associations between ERP measures and ADHD and autism, the effect sizes are small which may limit their predictive value. However, these correlations, remain important despite their small size because they contribute to the understanding of aetiological mechanisms of these disorders. Both ADHD and autism are likely to be determined by numerous factors, each of which makes only a small contribution, which therefore may result in small effect sizes. Considering this, we believe none of these measures will be sufficient for diagnosis of ADHD and autism but could well be considered as aetiologically important in the development of the disorders alongside other measures.

A central aim of the study was to investigate associations between ERP indices of the CPT‐OX and WFIRS and QoL. While the CPT‐OX ERPs were broadly related to functional impairment and QoL, evidence for genetic/environmental overlap was limited. Impairment in work adjustments was phenotypically associated with the greatest number of ERP factors (FA1, FA3 and FA5, Figure 2) indicating widespread association between this measure and ERP measures. Yet, no evidence emerged for shared genetic aetiology. Family relationships also showed an association with FA5. These findings indicate some impact of the timing of preparatory signals on functional impairment in both work and family domains. Despite the strong phenotypic and genetic relationships between work adjustment and ADHD, the lack of significant associations of FA1 or FA5 with ADHD and the unique influence evidenced by path estimates between these factors and work adjustment (Table S4) suggests a relationship between these ERP measures and the ability to work over and above the ADHD diagnosis. Social relationships (QoL) showed negative relationships with both FA2 and FA6: source cue events (N2 and P3, central and parietal‐occipital). These findings contrast with the findings for social functioning (WFIRS), which showed phenotypic correlation only with FA4 (source no‐go P3). An explanation for this distinction could be related to administration of the QoL and WFIRS: the QoL depends on the individual's own perception with a subjective threshold, whereas functional impairments assessed by the examiner (WFIRS) have more objective thresholds via the comparison with other individuals (Danckaerts et al., 2010).

### Limitations

Despite having one of the largest cohorts of ADHD and autistic individuals with EEG recordings, we are limited in our ability to detect significant genetic and environmental contributions due to sample size. This is especially the case when phenotypic correlation is significant but none of the separate contributions of genetics, common environment or unique environment (and error) were significant. For example, despite significant phenotypic correlations and substantial genetic associations between autism and physical health (94% and a significant Ra = −0.28) and psychological (85%) QoL, the genetic contributions were not statistically significant, which may be due to the sample being underpowered or it is also possible that this is due to lack of a true effect. The relatively small sample size for a twin study required reduction of the number of tests performed. To find a balance between potential type I and type II errors, variables were preselected for investigation in the twin modelling using multilevel mixed effects models testing for phenotypic relationships (independent of twin relatedness). However, the selection of variables may necessarily exclude information about additional alterations in brain function in the conditions. Since the study sample was enriched for high levels of autistic and/or ADHD traits, we fixed prevalence thresholds and heritability to population values based on representative twin samples. As the estimates are not from the exact same population, but from similar populations, there may be minor influences on the model estimates.

While the WFIRS was originally proposed to measure functional impairment for adults with ADHD, which might be used to explain the lack of associations with autism for some domains, this is unlikely given the clear relevance of WFIRS domains for autism. We were unable to investigate comorbid ADHD and autism as a separate group due to the low number of cases with comorbid diagnosis in our sample. It would be of interest to examine the comorbid diagnosis in young adulthood in the future given the evidence for an additive model of cognitive impairments for the comorbid group in childhood (Tye et al., 2014).

## CONCLUSIONS

To date, this is the largest study of ADHD and autism using portable EEG and shows the feasibility of such data collection, which may open up future research to include participants who are unable to travel to a research centre (Lau‐Zhu, Lau, et al., 2019). Both ADHD and autism are associated with a decrease in QoL with strong genetic contributions specific to ADHD. ADHD was associated with increased functional impairment across all domains while autism was specifically associated with impaired social functioning and lower impairment in risk‐taking. Alterations in ERP measures from the CPT‐OX, particularly in relation to inhibitory and preparatory processing were associated with ADHD/autism and also with WFIRS and QoL. These findings align with robust and consistent patterns of cognitive alterations emerging in the literature on ADHD and autism, and could represent a step towards identifying measures that supplement the diagnosis with specific vulnerabilities in cognition and brain function (Insel et al., 2010; McLoughlin, Makeig, & Tsuang, 2014). The use of ERP measures along with other measures including facets of executive functioning to predict and track outcomes – for example, education, physical health, emotional and adaptive functioning ‐ may have greater clinical impact than a focus on diagnosis alone (McLoughlin et al., 2022).

## AUTHOR CONTRIBUTION

**Ümit Aydin**: Conceptualization; Data curation; Formal analysis; Investigation; Methodology; Software; Validation; Visualization; Writing – original draft; Writing – review & editing. **Simone J. Capp**: Data curation; Investigation; Writing – review & editing. **Charlotte Tye**: Writing – review & editing. **Emma Colvert**: Investigation; Writing – review & editing. **Alex Lau‐Zhu**: Investigation; Writing – review & editing. **Frühling Rijsdijk**: Methodology; Software; Writing – review & editing. **Jason Palmer**: Conceptualization; Methodology; Supervision; Writing – review & editing. **Gráinne McLoughlin**: Conceptualization; Funding acquisition; Methodology; Project administration; Resources; Supervision; Validation; Writing – review & editing.

## CONFLICTS OF INTEREST

The authors have declared that they have no competing or potential conflicts of interest.

## ETHICAL CONSIDERATIONS

Full ethical approval for the study was received from King's College London Psychiatry, Nursing and Midwifery Research Ethics Subcommittee (RESCMR‐16/17–2673).

## Supporting information

Supporting Information S1

## Data Availability

Available from the corresponding author upon reasonable request.
