# Supplementary material for: Quality of life, functional impairment and continuous performance task event‐related potentials (ERPs) in young adults with ADHD and autism: A twin study
Source: JCPP Adv. 2022 Jul 10;2(3):e12090. doi: 10.1002/jcv2.12090 (PMC10242939; doi:10.1002/jcv2.12090)
Supplement: Supplementary file 1 — Supporting Information S1 [file JCV2-2-e12090-s001.docx]

# **SUPPORTING INFORMATION**

**Quality of life, functional impairment and continuous performance task event-related potentials (ERPs) in young adults with ADHD and autism: a twin study**

**Ümit Aydin, Simone J. Capp, Charlotte Tye, Emma Colvert, Alex Lau-Zhu, Frühling Rijsdijk, Jason Palmer,** Gráinne McLoughlin

**MATERIALS AND METHODS**

**Sample**

All participants signed written informed consent prior to participation. Data were collected as part of the Individual Differences in EEG in young Adults Study (IDEAS) from February 2017 to May 2019. IDEAS aimed to recruit 600 participants (from 300 twin pairs) to establish a young adult sample enriched for autistic and ADHD traits. In total, 1,144 participants were invited to take part in the study across the four recruitment routes. Of these, 566 participants (271 males) with an average age of 22.44±0.96 years agreed to take part in the study. In ten twin pairs, data was missing for one twin; therefore, the final number of participants was 556 (119 Monozygotic (MZ) and 164 Dizygotic (DZ) pairs). Details of the recruitment is also available in (Capp et al., Submitted).

The medical exclusions were same as those used in the Twins Early Development Study (TEDS) (Haworth et al., 2013). These exclusions were conditions in the following categories: (1) severe ASD (Cases that are non-verbal or with severely delayed speech, or with difficulties in completing activities.); (2) severe cerebral palsy; (3) chromosomal disorders; (4) inherited or single-gene disorders associated with mental impairment; (5) brain damage or disorders affecting brain function; (6) profound deafness or complete blindness; (7) global developmental delay. More details on these exclusions could be found in related TEDS webpage (*TEDS Exclusions*, n.d.).

The four recruitment routes followed were:

**The Social Relationships (SR) Study-Phase 1.** The SR Study is an established subsample of TEDS focusing on twin pairs where one or both twins met autism diagnostic criteria or displayed a subclinical autism phenotype in adolescence (described in (Colvert et al., 2015)). There have been three data collection phases of the SR Study, the latest of which was completed concurrently with IDEAS. Twins were initially screened for invitation to the SR Study Phase 1 using the Childhood Autism Spectrum Test (CAST; (Williams et al., 2005)) at age 8 (parent reports). If parents endorsed 15 or more items on the CAST for either twin, they were considered to have a high likelihood of at least one twin meeting autism spectrum diagnostic criteria. Subsequently, parents were asked to complete the Development and Well-Being Assessment (DAWBA; (Goodman et al., 2000)) by telephone. Families were then invited to take part in the SR study if one or both twins met autism spectrum diagnostic thresholds based on the DAWBA.

Control families were recruited from TEDS based on low CAST scores (both twins <12) and matched on several variables to the SR Study autism group. The SR Study employed a multistage research diagnosis process and participants completed a wide range of cognitive and behavioural tasks, including IQ tests with the Wechsler Abbreviated Scale of Intelligence (Wechsler, 1999).

A total of 290 participants from the SR Study Phase 1 sample were contacted by IDEAS (126 autism group, 50 co-twins, 114 controls). Of these, 92 agreed to participate in IDEAS (26 autism group, 14 co-twins, 52 controls). 48 participants from the SR study sample were not invited to take part in IDEAS due to time restraints. Families where either twin was estimated to have an IQ <70 from the SR Study Phase 1 in adolescence were not invited to take part in IDEAS (N=82 excluded). Due to the demands of the various questionnaires, interviews, and behavioural tasks in IDEAS, it was thought that those with an IQ <70 would not be able to understand and complete all assessments. While it is possible that some individuals with a recorded IQ<70 in adolescence could have made considerable gains by young adulthood to enable them to take part in the study, IQ has been demonstrated to show high stability across this timespan (Deary et al., 2000). Therefore, it is unlikely that a large number of families where twins truly did have capacity would have been excluded in this way.

**The Neurophysiological Study of Activity and Attention in Twins Study (NEAAT).** NEAAT recruited and tested a subsample of male TEDS twins at age 14 based on ADHD symptoms (Tye et al., 2012). Scores on the Revised Conners Parent Rating Scale (Conners et al., 1998) at multiple time points between the ages of 8-14 years were used in longitudinal latent class analyses to identify individuals with stably low, intermediate or high ADHD scores. Twin pairs where one or both twins were grouped into the high ADHD class were invited to take part in NEAAT. A control group was also recruited to NEAAT from families where both twins were classed as having stably low ADHD traits. A total of 130 participants from the NEAAT sample were contacted by the IDEAS team (49 ADHD,8 low traits co-twins,73 comparison twins). Of these, 62 were successfully contacted and agreed to participate in IDEAS team (21 ADHD, 3 low traits co-twins, 38 comparison twins). A further 4 participants from the NEAAT sample were not invited to take part in IDEAS.

**The SR Study-Phase 3.** The SR Study undertook a third phase of data collection when the twins were aged 20-25. As a part of the research design, additional participants, not from the original SR Study Phase 1 sample were recruited from TEDS. This was to include participants who had since reported an autism spectrum diagnosis or had high autistic traits but no diagnosis. Sixty-four participants from the SR Study Phase 3, who had not taken part in Phase 1 or NEAAT, were invited to take part in IDEAS. Of these, 48 participants participated. All these participants had a formal autism spectrum diagnosis (N=15), or high traits (N=33) as rated by the study team based on multiple current and past measures.

**Main TEDS cohort.** Participants recruited from the SR Study included male, female and opposite sex pairs. However, participants recruited from NEAAT were all male. Therefore, further attempts were made to recruit female and opposite sex ‘ADHD’ twin pairs from the main TEDS cohort. Latent class models initially used to select NEAAT participants were replicated including female and opposite sex pairs and additional participants with stably high ADHD traits at ages 8-14 were identified. These participants were invited to take part in IDEAS if they had an estimated IQ > 70 from TEDS records. Additional control participants were identified from the main TEDS cohort and invited to take part in IDEAS. These twin pairs were selected if both showed stably low ADHD symptoms from latent class analyses, had low age 8 CAST scores (<12), and estimated IQ > 70. A total of 660 participants were invited to IDEAS directly from the mains TEDS cohort. Of these, 354 went on to take part.

**Data cleaning and error correction**

All data from in-person measures were entered twice by two different members of the team. The compare datasets function in SPSS (IBM Corp, 2016) was used to identify discrepancy between the entries. Discrepancies were evaluated against the original hard copies and corrected in both datasets. Datafiles from in-person assessments and online questionnaires were merged using SPSS. An iterative procedure involving the compare datasets function, manual checks and reviewing participant records was carried out to ensure that each participant was uniquely and correctly identified by a single ID code which matched existing TEDS records. Common errors corrected included participants entering their twin’s ID code in online questionnaires, and mistyped or transposed digits in ID codes. Details of this process can be found in supplementary materials of (Capp et al., Submitted)

**Missing data/multiple imputation**

The number of participants with valid psychological measures and EEG measurements are shown in (Suppl. Table 1). Missing data were handled using Multiple Imputation (MI) using Chained Equations - MICE package in R (Buuren & Groothuis-Oudshoorn, 2011). We used imputed data only for the mixed effects model *and not for the twin modelling* since OpenMx uses full-information maximum-likelihood (FIML) which is robust to missingness at random (Schafer & Graham, 2002). Five imputations were created with multiple imputation and results were analysed. In addition to the DIVA-2, ADOS-2, WFIRS, QoL and EEG measures, age, sex and four questionnaire measures were used in the multiple imputation. The four online questionaries that were only used in multiple imputation were:

*-* ***Mill Hill Vocabulary Scale and Ravens Standard Progressive Matrices*** (Raven et al., 1998)*:* were used to create a general measure for cognitive ability composite score following the procedure in Haworth et al. (Haworth et al., 2010).

*-* ***Barkley Adult ADHD Rating Scale-IV (BAARS):*** is an empirically developed self-rating scale, based on DSM diagnostic criteria, that evaluates the most reliable underlying dimensions of the symptom list for adults (Barkley, 2011).

- ***Social Responsiveness Scale 2nd edition (SRS-2):*** is a self-reported scale measuring deficit in social behaviour associated with autism and their severity (Constantino & Gruber, 2012).

***World Health Organization Quality of Life-BREF (QoL):*** The BREF asks participants to reflect on their life in the last two weeks and each item has a 5-point like Likert response scale. The physical health domain (7 items) includes questions on physical pain, sleep, and energy for everyday activities. The psychological domain (6 items) includes positive and negative feelings, and personal beliefs. The social relationships domain (3 items) covers personal relationships, social support, and sex life. Finally, the environmental domain (8 items) includes financial resources, home environment, and opportunities for leisure activities. For each domain possible scores range from 4-20 with higher scores indicating higher QoL (The WHOQOL Group, 1998).

**EEG analysis**

For the independent component analysis (ICA) raw data was filtered within the band 1Hz to 30Hz and resampled to 256Hz. Bad channels were removed automatically based on extended periods of low correlation (<0.4) with neighbouring channels (McLoughlin et al., 2018) or extended periods of high amplitudes (>75μV absolute value). After deleting the bad channels, EEG data was re-referenced to average reference and intervals with an EEG amplitude higher than the mean plus five times the standard deviation of the EEG run were marked as bad time-intervals. Following the bad channel and time interval removal Adaptive Mixture ICA (AMICA) was used to calculate the ICA components (Palmer et al., 2011). We used the EEGLAB nsgportal plug-in to calculate AMICA on the high performance computing available on The Neuroscience Gateway (NSG, nsgportal.org) (Martínez-Cancino et al., 2021).

Once the ICA weights were calculated from the 1Hz high-pass filtered data these were applied to the original EEG data that was filtered within the band 0.1Hz to 30Hz, resampled to 256Hz and average referenced. Equivalent current dipoles were calculated for each IC component with a template four-layer boundary element method head model using dipfit function in EEGLAB (Oostendorp & van Oosterom, 1989). Dipoles located outside the brain or with higher than 15% residual variance were rejected. The Eyecatch algorithm, which automatically detects the ocular ICA components based on their scalp maps and spectrums, was used for the detection and removal of ocular artefacts (Bigdely-Shamlo et al., 2013). On average 3.04 ICs were rejected per subject with a standard deviation of 2.26. A mixed effects model indicated the number of rejected components was not related to diagnosis.

Continuous data were epoched -500 to 1650ms around the cue, go, and no-go events, baseline corrected using the prestimulus interval -500 to -200ms, and bad epochs exceeding an amplitude threshold (±150uV) were removed.

Signals measured at EEG channels at the scalp surface is a summation of activity from many cortical sources and weights of this summation depends on the volume conduction. It is possible to estimate the source-based signals (cortical activity) from the activity measured with EEG using source localisation methods. Source-based signals coupled with ICA have been shown to share more genetic variance with behaviour than channel-based EEG measures (McLoughlin et al., 2014). Thus, we studied both channel-based and source-based activity. We used measure projection toolbox available in EEGLAB with default parameters to calculate source-based activity. ERP waveforms and dipole locations were used for creating the domains. We were unable to identify a corresponding source-based measure for the CNV, possibly due to its low frequency; therefore only the channel-based measure was used. Latency windows were selected based on visual investigation of the individual and group level signals to ensure they are wide enough to capture the inherent individual variability of the peak latencies while being narrow enough to ensure distinction in peaks selected from different components.

**Selection of variables in phenotypic analysis**

EEG measures and WFIRS/QoL variables were preselected for investigation in the twin modelling using multilevel mixed effects models to test for phenotypic (independent of twin relatedness) relationships. Only the variables related with each other with a trend of p<0.1 in the phenotypic analysis were included in the genetic models. Age and sex were accounted for as covariates and a random intercept was used to control for twin relatedness (Malone et al., 2014). To include all data in the phenotypic selection analysis, the MICE package in R (Buuren & Groothuis-Oudshoorn, 2011) was used for multiple imputation (5 imputations) and lme4 and lmerTest packages were used for the mixed effects model (Bates et al., 2015; Kuznetsova et al., 2017). Imputed data were only used for the mixed effects model and not for the twin modelling since OpenMx uses full-information maximum-likelihood (FIML) estimation which is robust to missingness at random (Schafer & Graham, 2002). All measures with skewed distributions were normalised using Box-Cox transformation prior to analysis.

**Twin modelling**

Three different models were fitted on raw data of the whole sample, including typically developed (TD) individuals (Suppl. Figure 1). The first model estimated the phenotypic correlation between ADHD and autism using liability thresholds for both ADHD and autism with fixed twin correlation and model parameters for both conditions (detailed above and in Suppl. Figure 1a). The second bivariate model included ADHD or autism (with liability threshold) and one ERP factor, WFIRS or QoL measure (Suppl. Figure 1b). Due to convergence problems, a trivariate model with both ADHD and autism was not possible, likely due to the use of liability thresholds for both ADHD and autism in the same model. The third model included ADHD or autism, an ERP factor, and WFIRS or QoL for the purpose of investigating the associations between ERP measures and WFIRS or QoL measure. It was necessary to include ADHD and autism variables to avoid bias due to the enriched study sample. The phenotypic correlations that were estimated between ADHD or autism and the ERP factors or questionnaires with the trivariate model were very similar to the ones reported with bivariate models (Tables 1 and 2), and thus, are not reported again in Table 3.

**SUPPLEMENTARY RESULTS**

***The phenotypic correlation between ADHD and autism for young adults and the role of genetics in this phenotypic correlation***

In agreement with previous studies, we found a statistically significant phenotypic correlation between ADHD and autism for young adults at 0.211 (CI: 0.043;0.377). The model estimated a genetic contribution of 87% (0.184, CI: ‑0.027;0.391) and 13% (0.028, CI: -0.135;0.187) due to unique environment but the genetic and environment contribution estimates failed significance. This could be due to use of the strict diagnostic criteria of the ADOS-2 and the DIVA-2 rather than trait measures commonly used in twin studies of autism and ADHD (Ghirardi et al., 2019; Reiersen et al., 2008).

**Findings when co-occurring ADHD-autism cases are excluded from the analysis**

Findings of the bivariate twin models to investigate the heritability of QoL, WFIRS, and EEG factors and the association of these measures to ADHD and autism when co-occurring cases were excluded are presented in Suppl. Table 2 and 3. Values showing a change in significance in comparison to Table 1 and 2 are highlighted with red font in Suppl. Table 2 and 3. The majority of the differences are observed for associations with autism. Phenotypic correlations between autism and QoL-1 (physical health), QoL-2 (psychological), QoL-4 (environment), WFIRS domain and total mean as well as genetic correlations between autism and WFIRS social and WFIRS risk became not significant when co-occurring cases were excluded. In the case of ADHD, its phenotypic correlation with QoL-3 (social relationships) became not significant.

When co-occurring cases were excluded, previously significant genetic contributions (both Rph-a and Ra) between FA3 and ADHD as well as FA6 and autism were no longer significant. Further, the genetic contributions between FA7 and autism became significant when co‑occurring cases were excluded. In addition, the phenotypic correlations between FA3 and autism and FA2 and ADHD reduced so that the lower threshold of the confidence interval was close to zero. Similarly, the genetic contributions to the phenotypic correlation (Rph-a) between FA3 and autism was substantially reduced despite the genetic correlations (Ra) remaining significant. These results should be interpreted with caution as the differences observed between co-occurring cases included and excluded might be heavily influenced by the changes in the number of ADHD and autism cases. The number of ADHD cases reduced from 111 to 93 (16% decrease; in addition to 16 co-occuring, 2 participants with ADHD but without ADOS-2 scores were also excluded) but the number of autism cases reduced from 47 to 31 (34% decrease) when co-occurring cases were excluded. Therefore, it is possible that the observed differences are due to the reduced number of cases, especially in the autism group, and not due to the overlap of ADHD and autism.

**Table S1:** Number of participants that completed each psychological measure and the number of participants with valid ERP measures after artefact rejection. * Cognitive ability calculated from Mill Hill Vocabulary Scale and Ravens Standard Progressive Matrices

| Psychological and EEG measures | Number of participants with valid data prior to multiple imputation |
| --- | --- |
| DIVA-2 | 556 |
| ADOS-2 | 547 |
| SRS-2 | 483 |
| BAARS | 490 |
| Cognitive ability* | 494 |
| QOL | 482 |
| WFIRS Family | 473 |
| WFIRS Work | 407 |
| WFIRS School | 268 |
| WFIRS Life Skills | 471 |
| WFIRS Self Concept | 471 |
| WFIRS Social | 469 |
| WFIRS Risk | 469 |
| WFIRS Domains mean | 473 |
| WFIRS Total mean | 473 |
| ERP measures | 509 |

**Table S2:** Findings of the bivariate twin model to investigate the heritability of QoL and WFIRS and the association of these measures to ADHD and autism.

| **Measures** | **Condition** | **rMZ** | **rDz** | **a^2^** | **c^2^** | **e^2^** | **Rph-total** | **Rph-a** | **Rph-e** | **Ra** |
| --- | --- | --- | --- | --- | --- | --- | --- | --- | --- | --- |
| **QoL-1**  **Physical health** | **ADHD** | **0.54**  0.40,0.64 | **0.25**  0.07,0.41 | **0.53**  0.21,0.64 | 0.00  0.00,0.26 | **0.47**  0.36,0.60 | **-0.29**  -0.40,-0.18 | **-0.14**  -0.28,-0.01 | **-0.15**  -0.25,-0.04 | **-0.22**  -0.44,-0.01 |
|  | **Autism** | **0.53**  0.39,0.64 | **0.27**  0.09,0.43 | **0.52**  0.11,0.64 | 0.01  0.00,0.35 | **0.47**  0.36,0.61 | -0.07  -0.22,0.09 | -0.08  -0.28,0.12 | 0.01  -0.17,0.19 | -0.13  -0.55,0.22 |
| **QoL-2**  **Psychological** | **ADHD** | **0.50**  0.35,0.62 | **0.23**  0.03,0.40 | **0.49**  0.10,0.61 | 0.00  0.00,0.32 | **0.51**  0.39,0.65 | **-0.22**  -0.32,-0.11 | **-0.18**  -0.31,-0.05 | -0.04  -0.15,0.07 | **-0.30**  -0.52,-0.08 |
|  | **Autism** | **0.51**  0.36,0.62 | **0.24**  0.03,0.41 | **0.50**  0.10,0.62 | 0.00  0.00,0.33 | **0.50**  0.38,0.63 | -0.07  -0.22,0.09 | -0.08  -0.30,0.15 | 0.01  -0.20,0.22 | -0.13  -0.57,0.25 |
| **QoL-3**  **Social relationships** | **ADHD** | **0.21**  0.02,0.38 | 0.19  0.00,0.36 | *0.05*  *0.00,0.38* | *0.16*  *0.00,0.32* | **0.79**  0.62,0.93 | -0.09  -0.20,0.03 | -0.04  -0.18,0.10 | -0.05  -0.18,0.08 | -0.21  -1.00,1.00 |
|  | **Autism** | **0.21**  0.03,0.38 | 0.19  0.00,0.37 | *0.04*  *0.00,0.38* | *0.17*  *0.00,0.33* | **0.79**  0.62,0.93 | **-0.18**  -0.33,-0.03 | -0.05  -0.26,0.16 | -0.14  -0.34,0.10 | -0.28  -1.00,1.00 |
| **QoL-4 Environment** | **ADHD** | **0.45**  0.30,0.56 | **0.25**  0.04,0.43 | **0.41**  0.01,0.56 | 0.03  0.00,0.40 | **0.56**  0.44,0.70 | **-0.19**  -0.30,-0.07 | **-0.17**  -0.30,-0.03 | -0.02  -0.14,0.09 | **-0.30**  -1.00,-0.05 |
|  | **Autism** | **0.45**  0.31,0.57 | **0.27**  0.06,0.44 | *0.35*  *0.00,0.57* | *0.10*  *0.00,0.45* | **0.55**  0.43,0.69 | -0.12  -0.27,0.04 | -0.08  -0.29,0.13 | -0.04  -0.24,0.18 | -0.15  -1.00,1.00 |
| **WFIRS Family relations** | **ADHD** | **0.41**  0.23,0.55 | 0.06  -0.12,0.24 | **0.36**  0.09,0.50 | 0.00  0.00,0.19 | **0.64**  0.50,0.81 | **0.32**  0.21,0.42 | **0.31**  0.17,0.44 | 0.01  -0.11,0.14 | **0.60**  0.33,1.00 |
|  | **Autism** | **0.44**  0.26,0.57 | 0.10  -0.10,0.28 | **0.40**  0.11,0.54 | 0.00  0.00,0.21 | **0.60**  0.46,0.77 | 0.05  -0.11,0.21 | -0.15  -0.36,0.08 | 0.20  -0.03,0.38 | -0.28  -0.76,0.15 |
| **WFIRS Work adjustments** | **ADHD** | **0.30**  0.10,0.48 | 0.18  -0.07,0.40 | **0.25**  0.01,0.47 | 0.06  0.00,0.34 | **0.70**  0.53,0.88 | **0.37**  0.26,0.48 | **0.24**  0.10,0.38 | 0.13  -0.01,0.26 | **0.56**  0.20,1.00 |
|  | **Autism** | **0.31**  0.10,0.48 | 0.23  -0.03,0.44 | *0.17*  *0.00,0.49* | *0.15*  *0.00,0.42* | **0.69**  0.51,0.87 | 0.12  -0.08,0.30 | -0.04  -0.28,0.22 | 0.15  -0.14,0.42 | -0.10  -1.00,1.00 |
| **WFIRS School performance** | **ADHD** | **0.52**  0.26,0.69 | **0.43**  0.15,0.62 | *0.29*  *0.00,0.69* | *0.24*  *0.00,0.56* | **0.47**  0.31,0.70 | **0.41**  0.28,0.53 | **0.21**  0.05,0.36 | **0.20**  0.07,0.32 | **0.45**  0.10,1.00 |
|  | **Autism** | **0.51**  0.24,0.68 | **0.44**  0.16,0.64 | *0.16*  *0.00,0.68* | *0.36*  *0.00,0.60* | **0.48**  0.31,0.69 | -0.03  -0.28,0.20 | -0.24  -0.51,0.13 | 0.20  -0.22,0.40 | -0.67  -1.00,1.00 |
| **WFIRS Life skills** | **ADHD** | **0.46**  0.29,0.59 | 0.15  -0.04,0.32 | **0.44**  0.12,0.57 | 0.00  0.00,0.23 | **0.56**  0.43,0.72 | **0.38**  0.27,0.48 | **0.27**  0.14,0.40 | 0.11  -0.01,0.22 | **0.47**  0.24,0.94 |
|  | **Autism** | **0.47**  0.30,0.60 | 0.17  -0.02,0.34 | **0.46**  0.12,0.59 | 0.00  0.00,0.26 | **0.54**  0.41,0.69 | 0.04  -0.12,0.20 | -0.03  -0.25,0.19 | 0.08  -0.14,0.26 | -0.05  -0.47,0.32 |
| **WFIRS Self‑concept** | **ADHD** | **0.52**  0.38,0.64 | **0.23**  0.05,0.40 | **0.52**  0.15,0.63 | 0.00  0.00,0.29 | **0.48**  0.37,0.62 | **0.25**  0.14,0.36 | **0.18**  0.05,0.32 | 0.06  -0.04,0.17 | **0.29**  0.08,0.64 |
|  | **Autism** | **0.53**  0.38,0.64 | **0.25**  0.06,0.42 | **0.52**  0.12,0.64 | 0.00  0.00,0.33 | **0.48**  0.36,0.62 | 0.00  -0.16,0.16 | -0.01  -0.22,0.21 | 0.01  -0.18,0.19 | -0.02  -0.41,0.35 |
| **WFIRS Social functioning** | **ADHD** | **0.55**  0.39,0.66 | 0.06  -0.13,0.24 | **0.49**  0.27,0.62 | 0.00  0.00,0.14 | **0.51**  0.38,0.68 | **0.29**  0.17,0.39 | **0.21**  0.07,0.35 | 0.07  -0.04,0.18 | **0.35**  0.12,0.58 |
|  | **Autism** | **0.55**  0.40,0.67 | 0.07  -0.13,0.25 | **0.50**  0.28,0.63 | 0.00  0.00,0.14 | **0.50**  0.37,0.66 | **0.18**  0.02,0.33 | 0.04  -0.16,0.25 | 0.14  -0.06,0.30 | 0.06  -0.28,0.40 |
| **WFIRS Risk taking** | **ADHD** | **0.55**  0.41,0.66 | 0.06  -0.14,0.25 | **0.51**  0.29,0.63 | 0.00  0.00,0.15 | **0.49**  0.37,0.64 | **0.38**  0.27,0.48 | **0.24**  0.12,0.37 | **0.13**  0.03,0.23 | **0.39**  0.19,0.60 |
|  | **Autism** | **0.57**  0.43,0.68 | 0.02  -0.19,0.23 | **0.53**  0.33,0.65 | 0.00  0.00,0.13 | **0.47**  0.35,0.62 | -0.15  -0.31,0.01 | -0.11  -0.36,0.13 | -0.04  -0.27,0.20 | -0.17  -0.57,0.21 |
| **WFIRS Domain mean** | **ADHD** | **0.60**  0.46,0.69 | 0.17  -0.02,0.35 | **0.57**  0.34,0.68 | 0.00  0.00,0.17 | **0.43**  0.32,0.56 | **0.44**  0.33,0.53 | **0.31**  0.18,0.44 | **0.13**  0.02,0.23 | **0.47**  0.27,0.67 |
|  | **Autism** | **0.61**  0.48,0.71 | 0.19  -0.01,0.37 | **0.59**  0.35,0.69 | 0.00  0.00,0.19 | **0.41**  0.31,0.54 | 0.09  -0.08,0.25 | -0.08  -0.28,0.14 | 0.17  -0.03,0.32 | -0.13  -0.44,0.20 |
| **WFIRS Total mean** | **ADHD** | **0.58**  0.44,0.68 | 0.15  -0.04,0.33 | **0.55**  0.33,0.66 | 0.00  0.00,0.16 | **0.45**  0.34,0.59 | **0.47**  0.36,0.56 | **0.32**  0.19,0.45 | **0.14**  0.03,0.24 | **0.50**  0.30,0.71 |
|  | **Autism** | **0.59**  0.46,0.69 | 0.17  -0.03,0.35 | **0.58**  0.34,0.68 | 0.00  0.00,0.19 | **0.42**  0.32,0.56 | 0.10  -0.07,0.25 | -0.10  -0.29,0.12 | 0.19  -0.01,0.33 | -0.15  -0.46,0.18 |

* For each questionnaire two bivariate models were used, one with ADHD and one with autism, and they are both included in the tables; **rMz:** MZ twin correlations; **rDZ:** DZ twin correlations. 95% confidence intervals (CIs) are included under each estimate and significant estimate are written in bold.

****** Point estimates shown in italic reflect the cases where the confidence interval was close to zero (showing 0.00 due to rounding, though not overlapping with zero), in some cases this might be due to limited statistical power in the model to identify the genetic and common environment contribution separately. These results should be interpreted with caution.

**Table S3:** Findings of the bivariate twin model to investigate the heritability of seven ERP factors and the association of these factors to ADHD and autism.

| **ERP Factors** | **Condition** | **rMZ** | **rDz** | **a^2^** | **c^2^** | **e^2^** | **Rph-total** | **Rph-a** | **Rph-e** | **Ra** |
| --- | --- | --- | --- | --- | --- | --- | --- | --- | --- | --- |
| **FA1** | **ADHD** | **0.34**  0.15,0.49 | **0.21**  0.04,0.36 | *0.25*  *0.00,0.49* | *0.08*  *0.00,0.36* | **0.66**  0.51,0.84 | -0.02  -0.13,0.09 | -0.04  -0.17,0.10 | 0.01  -0.11,0.13 | -0.08  -1.00,1.00 |
|  | **Autism** | **0.34**  0.15,0.49 | **0.20**  0.03,0.36 | *0.28*  *0.00,0.49* | *0.07*  *0.00,0.35* | **0.66**  0.51,0.84 | 0.08  -0.07,0.23 | 0.13  -0.09,0.34 | -0.05  -0.26,0.18 | 0.29  -1.00,1.00 |
| **FA2** | **ADHD** | **0.20**  0.01,0.37 | **0.20**  0.02,0.35 | *0.01*  *0.00,0.37* | *0.19*  *0.00,0.31* | **0.80**  0.63,0.93 | *0.11*  *0.00,0.22* | 0.08  -0.05,0.21 | 0.03  -0.09,0.15 | 0.86  -1.00,1.00 |
|  | **Autism** | 0.19  0.00,0.37 | **0.18**  0.01,0.34 | *0.06*  *0.00,0.38* | *0.15*  *0.00,0.30* | **0.79**  0.62,0.93 | -0.09  -0.24,0.07 | 0.13  -0.09,0.34 | -0.22  -0.43,0.03 | 0.64  -1.00,1.00 |
| **FA3** | **ADHD** | **0.53**  0.36,0.65 | **0.22**  0.06,0.36 | **0.51**  0.18,0.63 | 0.00  0.00,0.24 | **0.49**  0.37,0.64 | **-0.16**  -0.27,-0.05 | -0.11  -0.25,0.03 | -0.05  -0.16,0.06 | -0.18  -0.41,0.04 |
|  | **Autism** | **0.54**  0.38,0.65 | **0.22**  0.06,0.37 | **0.52**  0.19,0.64 | 0.00  0.00,0.25 | **0.48**  0.36,0.63 | -0.15  -0.29,0.00 | *-0.21*  *-0.42,0.00* | 0.07  -0.14,0.28 | **-0.34**  -0.73,-0.01 |
| **FA4** | **ADHD** | **0.51**  0.33,0.63 | **0.32**  0.17,0.45 | *0.38*  *0.00,0.63* | *0.13*  *0.00,0.43* | **0.49**  0.37,0.67 | **0.12**  0.01,0.23 | 0.11  -0.03,0.24 | 0.01  -0.10,0.12 | 0.20  -0.72,1.00 |
|  | **Autism** | **0.51**  0.34,0.64 | **0.31**  0.16,0.44 | **0.41**  0.04,0.64 | 0.11  0.00,0.40 | **0.48**  0.36,0.65 | 0.05  -0.10,0.20 | **0.26**  0.04,0.45 | *-0.20*  *-0.36,0.00* | **0.46**  0.08,1.00 |
| **FA5** | **ADHD** | 0.21  -0.01,0.39 | 0.06  -0.11,0.22 | *0.18*  *0.00,0.35* | *0.00*  *0.00,0.22* | **0.82**  0.65,1.00 | -0.02  -0.12,0.09 | -0.03  -0.17,0.11 | 0.02  -0.12,0.15 | -0.09  -1.00,1.00 |
|  | **Autism** | 0.21  0.00,0.39 | 0.07  -0.09,0.23 | *0.18*  *0.00,0.35* | *0.00*  *0.00,0.23* | **0.82**  0.65,1.00 | -0.05  -0.20,0.10 | -0.02  -0.24,0.19 | -0.03  -0.25,0.22 | -0.05  -1.00,1.00 |
| **FA6** | **ADHD** | **0.35**  0.14,0.51 | **0.31**  0.16,0.45 | *0.07*  *0.00,0.49* | *0.28*  *0.00,0.43* | **0.65**  0.49,0.79 | -0.07  -0.17,0.04 | -0.09  -0.22,0.05 | 0.02  -0.10,0.13 | -0.39  -1.00,1.00 |
|  | **Autism** | **0.34**  0.13,0.50 | **0.32**  0.17,0.45 | *0.04*  *0.00,0.48* | *0.29*  *0.00,0.42* | **0.67**  0.55,0.79 | 0.08  -0.08,0.24 | 0.18  -0.04,0.37 | -0.10  -0.29,0.13 | 1.00  -1.00,1.00 |
| **FA7** | **ADHD** | **0.32**  0.12,0.49 | 0.16  0.00,0.31 | *0.32*  *0.00,0.47* | *0.00*  *0.00,0.31* | **0.68**  0.53,0.85 | 0.01  -0.10,0.12 | -0.07  -0.21,0.07 | 0.08  -0.05,0.20 | -0.14  -1.00,0.14 |
|  | **Autism** | **0.33**  0.13,0.49 | ***0.16***  *0.00,0.31* | **0.33**  0.01,0.48 | 0.00  0.00,0.29 | **0.67**  0.52,0.84 | -0.03  -0.18,0.12 | **-0.22**  -0.41,-0.01 | 0.19  -0.03,0.37 | **-0.45**  -1.00,-0.02 |

**Suppl. Table 4:** Path estimates (not standardised) for the trivariate twin model with ADHD or autism, ERP factors and WFIRS or QoL.

| **Variables** | **Condition** | **a_21_** | **a_31_** | **a_32_** | **e_21_** | **e_31_** | **e_32_** |
| --- | --- | --- | --- | --- | --- | --- | --- |
| **FA7**  **QoL-1 Physical health** | **ADHD** | -0.08  -0.23,0.08 | **-0.27**  -0.40,-0.13 | -0.13  -0.72,0.72 | 0.10  -0.15,0.35 | -0.18  -0.36,0.01 | -0.14  -0.27,0.01 |
|  | **Autism** | -0.16  -0.35,0.04 | ***-0.20***  *-0.39,0.00* | -0.16  -0.77,0.77 | 0.11  -0.25,0.46 | 0.00  -0.30,0.30 | -0.16  -0.31,-0.02 |
| **FA2**  **QoL-3 Social relationships** | **ADHD** | 0.06  -0.09,0.20 | -0.08  -0.22,0.07 | 0.31  -0.61,0.61 | 0.15  -0.06,0.37 | -0.14  -0.37,0.10 | -0.11  -0.25,0.05 |
|  | **Autism** | 0.04  -0.16,0.22 | -0.03  -0.24,0.16 | 0.34  -0.61,0.61 | -0.09  -0.43,0.26 | *-0.39*  *-0.71,0.00* | -0.20  -0.44,0.01 |
| **FA6**  **QoL-3 Social relationships** | **ADHD** | -0.05  -0.19,0.09 | -0.08  -0.22,0.07 | 0.21  -0.51,0.51 | 0.02  -0.20,0.23 | -0.14  -0.37,0.09 | 0.06  -0.08,0.20 |
|  | **Autism** | **0.23**  0.04,0.41 | -0.04  -0.23,0.15 | 0.26  -0.53,0.53 | -0.23  -0.54,0.13 | -0.35  -0.67,0.01 | -0.04  -0.34,0.16 |
| **FA5**  **WFIRS Family relations** | **ADHD** | -0.04  -0.18,0.11 | **0.31**  0.17,0.45 | 0.21  -0.63,0.63 | 0.05  -0.21,0.29 | 0.03  -0.19,0.26 | 0.03  -0.11,0.17 |
|  | **Autism** | -0.01  -0.21,0.18 | -0.10  -0.28,0.10 | 0.18  -0.74,0.74 | -0.11  -0.47,0.29 | 0.25  -0.06,0.54 | 0.06  -0.12,0.27 |
| **FA1**  **WFIRS Work adjustments** | **ADHD** | -0.06  -0.20,0.09 | **0.31**  0.16,0.45 | -0.19  -0.59,0.59 | 0.06  -0.16,0.28 | 0.15  -0.09,0.38 | -0.09  -0.27,0.07 |
|  | **Autism** | 0.04  -0.15,0.23 | 0.04  -0.18,0.26 | -0.18  -0.70,0.70 | 0.06  -0.27,0.39 | 0.05  -0.37,0.43 | -0.06  -0.25,0.12 |
| **FA3**  **WFIRS Work adjustments** | **ADHD** | **-0.16**  -0.30,-0.02 | **0.31**  0.17,0.46 | 0.12  -0.43,0.43 | -0.09  -0.29,0.12 | 0.14  -0.10,0.38 | -0.11  -0.27,0.06 |
|  | **Autism** | **-0.23**  -0.40,-0.05 | 0.05  -0.17,0.27 | 0.06  -0.50,0.50 | 0.01  -0.27,0.31 | 0.03  -0.39,0.41 | -0.12  -0.28,0.05 |
| **FA5**  **WFIRS Work adjustments** | **ADHD** | -0.04  -0.18,0.11 | **0.30**  0.16,0.45 | 0.29  -0.60,0.60 | 0.06  -0.19,0.30 | 0.15  -0.09,0.38 | -0.04  -0.18,0.12 |
|  | **Autism** | -0.01  -0.20,0.18 | 0.01  -0.20,0.23 | 0.35  -0.70,0.70 | -0.10  -0.46,0.30 | 0.12  -0.29,0.48 | -0.02  -0.17,0.18 |
| **FA4**  **WFIRS Social functioning** | **ADHD** | 0.14  -0.01,0.29 | **0.31**  0.17,0.45 | 0.14  -0.69,0.69 | 0.00  -0.21,0.21 | 0.13  -0.08,0.33 | 0.10  -0.04,0.25 |
|  | **Autism** | **0.25**  0.06,0.43 | 0.18  0.00,0.37 | 0.13  -0.77,0.77 | **-0.37**  -0.63,-0.05 | 0.26  -0.07,0.54 | 0.29  0.03,0.69 |

* Additive‑genetics/unique‑environment path estimates between condition and ERP factor (**a_21_**/**e_21_**), condition and QoL or WFIRS (**a_31_**/**e_31_**), and ERP factor and QoL or WFIRS (**a_32_**/**e_32_**).

**
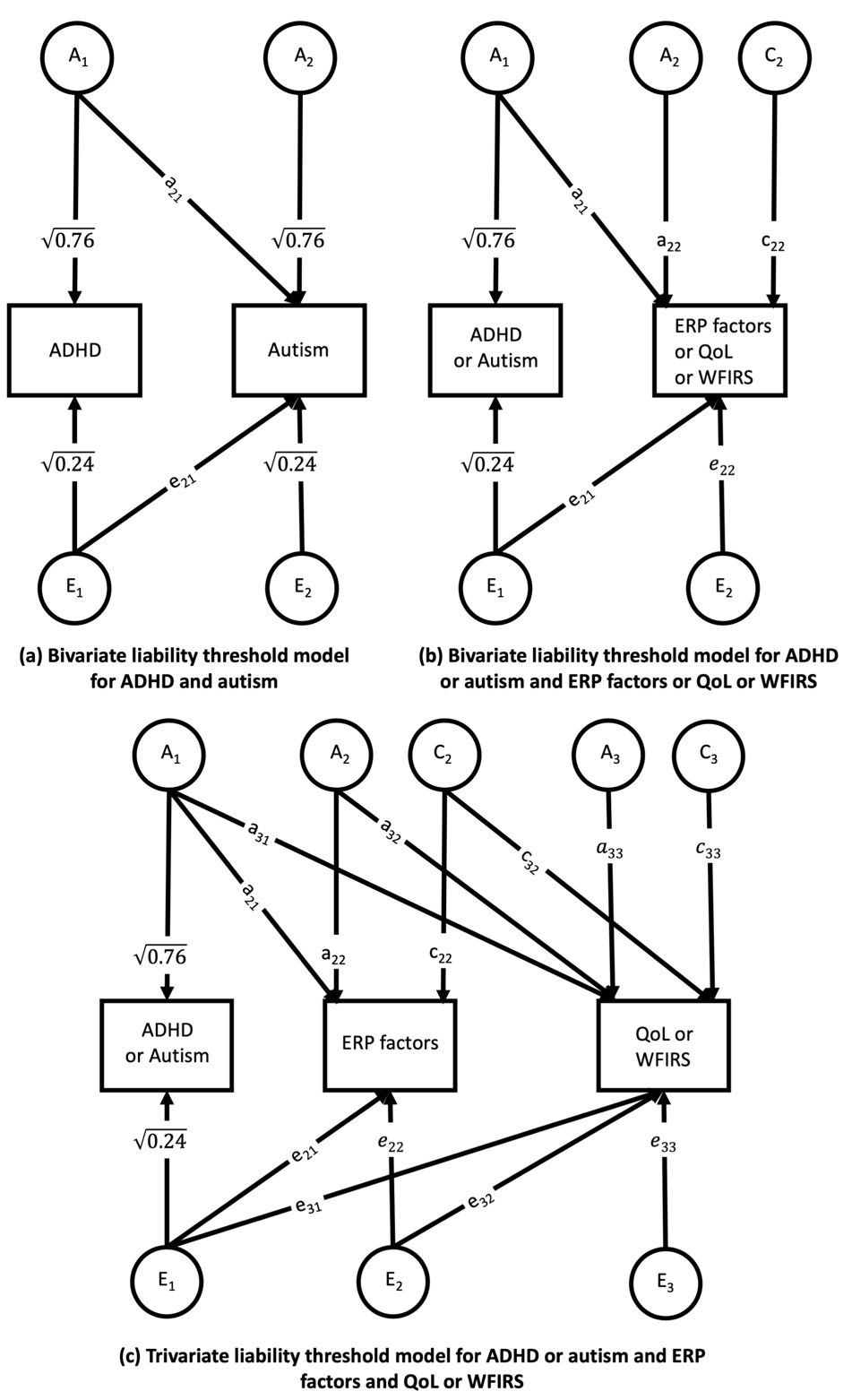
**

**Figure S1:** Schematics for the twin analysis. (a) Bivariate liability threshold model for ADHD and autism with fixed heritability parameters. (b) Bivariate model with liability threshold model for one of ADHD or autism with fixed heritability parameters and one of QoL or WFIRS or ERP factor. (c) Trivariate model with liability threshold model for one of ADHD or autism with fixed heritability parameters, one ERP factor and one of QoL or WFIRS.

## **REFERENCES**

Barkley, R. A. (2011). *Barkley Adult ADHD Rating Scale-IV (BAARS-IV)*. Guilford Press.

Bates, D., Mächler, M., Bolker, B., & Walker, S. (2015). Fitting Linear Mixed-Effects Models Using lme4. *Journal of Statistical Software*, *67*(1), 1–48. https://doi.org/10.18637/jss.v067.i01

Bigdely-Shamlo, N., Kreutz-Delgado, K., Kothe, C., & Makeig, S. (2013). EyeCatch: Data-mining over Half a Million EEG Independent Components to Construct a Fully-Automated Eye-Component Detector. *Conference Proceedings : ... Annual International Conference of the IEEE Engineering in Medicine and Biology Society. IEEE Engineering in Medicine and Biology Society. Conference*, *2013*, 5845–5848. https://doi.org/10.1109/EMBC.2013.6610881

Buuren, S. van, & Groothuis-Oudshoorn, K. (2011). mice: Multivariate Imputation by Chained Equations in R. *Journal of Statistical Software*, *45*(1), 1–67. https://doi.org/10.18637/jss.v045.i03

Capp, S., Agnew-Blais, J., Lau-Zhu, A., Colvert, E., Tye, C., Aydin, Ü., Lautarescu, A., Ellis, C., Saunders, T., O’Brien, L., Ronald, A., Happé, F., & McLoughlin, G. (Submitted). *Is Quality of Life Related to High Autistic Traits, High ADHD Traits and their Interaction? Evidence from a Young-Adult Population-Based Twin Sample*.

Colvert, E., Tick, B., McEwen, F., Stewart, C., Curran, S. R., Woodhouse, E., Gillan, N., Hallett, V., Lietz, S., Garnett, T., Ronald, A., Plomin, R., Rijsdijk, F., Happé, F., & Bolton, P. (2015). Heritability of Autism Spectrum Disorder in a UK Population-Based Twin Sample. *JAMA Psychiatry*, *72*(5), 415–423. https://doi.org/10.1001/jamapsychiatry.2014.3028

Conners, C. K., Sitarenios, G., Parker, J. D., & Epstein, J. N. (1998). The revised Conners’ Parent Rating Scale (CPRS-R): Factor structure, reliability, and criterion validity. *Journal of Abnormal Child Psychology*, *26*(4), 257–268. https://doi.org/10.1023/a:1022602400621

Constantino, J. N., & Gruber, C. P. (2012). *Social responsiveness scale: SRS-2*. Western Psychological Services Torrance, CA.

Deary, I. J., Whalley, L. J., Lemmon, H., Crawford, J. R., & Starr, J. M. (2000). The stability of individual differences in mental ability from childhood to old age: Follow-up of the 1932 Scottish Mental Survey. *Intelligence*, *28*(1), 49–55. https://doi.org/10.1016/S0160-2896(99)00031-8

Ghirardi, L., Pettersson, E., Taylor, M. J., Freitag, C. M., Franke, B., Asherson, P., Larsson, H., & Kuja-Halkola, R. (2019). Genetic and environmental contribution to the overlap between ADHD and ASD trait dimensions in young adults: A twin study. *Psychological Medicine*, *49*(10), 1713–1721. https://doi.org/10.1017/S003329171800243X

Goodman, R., Ford, T., Richards, H., Gatward, R., & Meltzer, H. (2000). The Development and Well-Being Assessment: Description and initial validation of an integrated assessment of child and adolescent psychopathology. *Journal of Child Psychology and Psychiatry, and Allied Disciplines*, *41*(5), 645–655.

Haworth, C. M. A., Davis, O. S. P., & Plomin, R. (2013). Twins Early Development Study (TEDS): A genetically sensitive investigation of cognitive and behavioral development from childhood to young adulthood. *Twin Research and Human Genetics: The Official Journal of the International Society for Twin Studies*, *16*(1), 117–125. https://doi.org/10.1017/thg.2012.91

Haworth, C. M. A., Wright, M. J., Luciano, M., Martin, N. G., de Geus, E. J. C., van Beijsterveldt, C. E. M., Bartels, M., Posthuma, D., Boomsma, D. I., Davis, O. S. P., Kovas, Y., Corley, R. P., Defries, J. C., Hewitt, J. K., Olson, R. K., Rhea, S.-A., Wadsworth, S. J., Iacono, W. G., McGue, M., … Plomin, R. (2010). The heritability of general cognitive ability increases linearly from childhood to young adulthood. *Molecular Psychiatry*, *15*(11), 1112–1120. https://doi.org/10.1038/mp.2009.55

Kuznetsova, A., Brockhoff, P. B., & Christensen, R. H. B. (2017). lmerTest Package: Tests in Linear Mixed Effects Models. *Journal of Statistical Software*, *82*, 1–26. https://doi.org/10.18637/jss.v082.i13

Malone, S. M., Burwell, S. J., Vaidyanathan, U., Miller, M. B., McGue, M., & Iacono, W. G. (2014). Heritability and Molecular-Genetic Basis of Resting EEG Activity: A Genome-Wide Association Study. *Psychophysiology*, *51*(12), 1225–1245. https://doi.org/10.1111/psyp.12344

Martínez-Cancino, R., Delorme, A., Truong, D., Artoni, F., Kreutz-Delgado, K., Sivagnanam, S., Yoshimoto, K., Majumdar, A., & Makeig, S. (2021). The open EEGLAB portal Interface: High-Performance computing with EEGLAB. *NeuroImage*, *224*, 116778. https://doi.org/10.1016/j.neuroimage.2020.116778

McLoughlin, G., Palmer, J. A., Rijsdijk, F., & Makeig, S. (2014). Genetic Overlap between Evoked Frontocentral Theta-Band Phase Variability, Reaction Time Variability, and Attention-Deficit/Hyperactivity Disorder Symptoms in a Twin Study. *Biological Psychiatry*, *75*(3), 238–247. https://doi.org/10.1016/j.biopsych.2013.07.020

McLoughlin, G., Palmer, J., Makeig, S., Bigdely-Shamlo, N., Banaschewski, T., Laucht, M., & Brandeis, D. (2018). EEG Source Imaging Indices of Cognitive Control Show Associations with Dopamine System Genes. *Brain Topography*, *31*(3), 392–406. https://doi.org/10.1007/s10548-017-0601-z

Oostendorp, T. F., & van Oosterom, A. (1989). Source parameter estimation in inhomogeneous volume conductors of arbitrary shape. *IEEE Transactions on Biomedical Engineering*, *36*(3), 382–391. https://doi.org/10.1109/10.19859

Palmer, J. A., Kreutz-Delgado, K., & Makeig, S. (2011). *AMICA: An Adaptive Mixture of Independent Component Analyzers with Shared Components*. 15.

Raven, J., Court, J. H., & Raven, J. C. (1998). *Manual for Raven’s progressive matrices and vocabulary scales*.

Reiersen, A. M., Constantino, J. N., Grimmer, M., Martin, N. G., & Todd, R. D. (2008). Evidence for shared genetic influences on self-reported ADHD and autistic symptoms in young adult Australian twins. *Twin Research and Human Genetics: The Official Journal of the International Society for Twin Studies*, *11*(6), 579–585. https://doi.org/10.1375/twin.11.6.579

Schafer, J. L., & Graham, J. W. (2002). Missing data: Our view of the state of the art. *Psychological Methods*, *7*(2), 147–177.

*TEDS Exclusions*. (n.d.). Retrieved February 7, 2022, from https://www.teds.ac.uk/datadictionary/exclusions.htm

The WHOQOL Group. (1998). Development of the World Health Organization WHOQOL-BREF Quality of Life Assessment. *Psychological Medicine*, *28*(3), 551–558. https://doi.org/10.1017/S0033291798006667

Tye, C., Rijsdijk, F., Greven, C. U., Kuntsi, J., Asherson, P., & McLoughlin, G. (2012). Shared genetic influences on ADHD symptoms and very low-frequency EEG activity: A twin study. *Journal of Child Psychology and Psychiatry, and Allied Disciplines*, *53*(6). https://doi.org/10.1111/j.1469-7610.2011.02501.x

Wechsler, D. (1999). *Wechsler Abbreviated Scale of Intelligence*. Psychological Corporation.

Williams, J., Scott, F., Stott, C., Allison, C., Bolton, P., Baron-Cohen, S., & Brayne, C. (2005). The CAST (Childhood Asperger Syndrome Test): Test accuracy. *Autism: The International Journal of Research and Practice*, *9*(1), 45–68. https://doi.org/10.1177/1362361305049029
